# Mitochondrial profiling across macrophage states reveals inhibition of IL-4/IL-13 reprogramming by the integrated stress response

**DOI:** 10.1126/sciadv.aed6318

**Published:** 2026-08-19

**Authors:** Joan Blanco-Fernandez, Miriam Lisci, Mads M. Foged, Chloé Chapuis, Tim Pflästerer, Tatjana Kleele, Alexis A. Jourdain

**Affiliations:** ^1^ Department of Immunobiology, Faculty of Biology and Medicine, University of Lausanne, Lausanne, Switzerland.; ^2^ Department of Biology, Institute of Biochemistry, ETH Zurich, Zurich, Switzerland.

## Abstract

Mitochondria drive cellular reprogramming by integrating metabolism and signaling. In macrophages, mitochondria are central to immunometabolic responses to external cues, but the extent to which they are remodeled and participate in macrophage reprogramming remains unclear. Here, we integrate transcriptomics with whole-cell and purified mitochondrial proteomics to profile lipopolysaccharide (LPS)/interferon-γ (IFN-γ)– and interleukin-4 (IL-4)/IL-13–stimulated macrophages. We reveal a notable disconnect between mitochondrial transcript and protein levels following either stimulus and a signal transducer and activator of transcription 6 (STAT6)–dependent increase in mitochondrial DNA (mtDNA) expression and intramitochondrial translation in IL-4/IL-13 macrophages. We demonstrate that pharmacological inhibition of mitochondrial translation or individual respiratory chain complexes variably impairs reprogramming, whereas ATP synthase inhibition uniquely triggers a heme-regulated inhibitor (HRI)–dependent integrated stress response (ISR) through mitochondrial hyperpolarization, thereby preventing IL-4/IL-13 reprogramming. Mechanistically, we show that restoring mitochondrial membrane potential or inhibiting the ISR rescues IL-4/IL-13–mediated reprogramming. Together, we identify mtDNA expression, intramitochondrial translation, and mitochondrial membrane potential as critical, drug-sensitive determinants of the IL-4/IL-13 response.

## INTRODUCTION

Mitochondria support cellular metabolism and signaling. Beyond producing adenosine 5′-triphosphate (ATP) through oxidative phosphorylation (OXPHOS), they contribute to key biosynthetic and signaling pathways that regulate gene expression, cellular fate, and adaptation (*1*, *2*). Mitochondrial dysfunction profoundly affects cellular function, and mitochondria have emerged as key determinants of immunity, as illustrated by the immune dysregulation and increased susceptibility to infection observed in patients with mitochondrial disorders (*3*, *4*).

Macrophages provide a powerful model to study mitochondria and metabolism in immunity owing to their plasticity in response to environmental cues and their essential role in inflammation, tissue homeostasis, and host defense. Distinct macrophage responses are associated with specific and sometimes contrasting metabolic programs. For example, whereas lipopolysaccharide (LPS) and interferon-γ (IFN-γ) dampen mitochondrial respiration and acutely promote glycolysis, interleukin-4 (IL-4) and IL-13 treatment promotes an increase in mitochondrial OXPHOS (*5*, *6*). Macrophage metabolic reprogramming is not merely a downstream consequence of their stimulation but also an active mediator of multiple functions, from cytokine secretion and immunometabolite synthesis to epigenetic reprogramming and effector protein expression (*6*–*11*).

Mitochondria lie at the core of the immunometabolic response, supporting energy production and metabolism and acting as signaling hubs. In LPS-stimulated macrophages, breaks in the tricarboxylic acid cycle lead to the accumulation of succinate and itaconate, two mitochondria-derived metabolites with immunomodulatory activity (*12*–*14*). Remodeling of the electron transport chain also increases the production of reactive oxygen species (ROS), amplifying cytokine secretion and bactericidal activity (*15*–*18*), while release of mitochondrial nucleic acids into the cytosol has been linked to activation of the inflammasome and the cytosolic DNA- and double-stranded RNA–sensing pathways (*19*–*22*). In contrast, less is known about the role of mitochondria in the response of macrophages to IL-4/IL-13 despite the characteristic increase in OXPHOS. Signal transducer and activator of transcription 6 (STAT6), the main transcription factor (TF) downstream of IL-4/IL-13 signaling, acts upstream of multiple TFs, including interferon regulatory factor 4 (IRF4), PGC-1β, and peroxisome proliferator–activated receptor γ (PPARγ), to support the IL-4/IL-13–mediated transcriptional and metabolic remodeling (*6*, *23*, *24*). However, the mechanism leading to the acute increase in respiration remains unclear. Furthermore, various mitochondria-targeted drugs have been reported to prevent the IL-4/IL-13 response in macrophages to varying extents (*6*–*8*, *25*, *26*), and many facets of mitochondrial dysfunction, including impaired oxidative ATP synthesis and metabolite production, nicotinamide adenine dinucleotide (NADH) accumulation, ROS signaling, and nucleic acid release, can potentially affect this response. The clinical manifestation of immune dysfunction and the susceptibility to infection in patients with mitochondrial disorders further highlight the importance of mitochondrial integrity for immune competence (*3*, *4*). However, to date, the mechanism through which mitochondrial dysfunction is signaled to the rest of the cell and how it prevents IL-4/IL-13–induced reprogramming remains unknown.

Macrophage responses to the environment have been extensively characterized using RNA sequencing (RNA-seq). However, transcriptional changes alone often fail to predict protein abundance, metabolic impact, and immune activity (*27*–*29*). While recent analyses of macrophage metabolism have described mitochondrial changes in response to pathogens and immunostimulatory molecules (*30*, *31*), proteomic studies of macrophages and their mitochondria are less common despite the importance of such studies in bridging the gap between transcription and metabolic changes upon stimulation. In addition, RNA-seq studies do not always include the transcripts encoded by the mitochondrial genome (*32*, *33*). The mammalian mitochondrial DNA (mtDNA) encodes 37 genes, 13 of which are translated within mitochondria and encode core subunits of the mitochondrial respiratory chain that are strictly required for OXPHOS. Mitochondrial ribosomes have a prokaryotic origin, which makes mitochondrial protein synthesis sensitive to several classes of prescribed antibiotics. Consistently, treatment with doxycycline or linezolid (LIN) has been shown to impair T lymphocyte function, suggesting that commonly prescribed antibiotics may interfere with aspects of immune cell function by blocking intramitochondrial protein synthesis (*34*, *35*).

Here, we integrate transcriptomics with proteomics analyses of whole cells or highly purified mitochondria to characterize mitochondrial remodeling in LPS/IFN-γ– and IL-4/IL-13–treated macrophages. We reveal an unexpectedly low correlation between mitochondrial transcripts and proteins upon macrophage activation and identify increased mtDNA expression among the most strongly up-regulated pathways in response to IL-4/IL-13. Using pharmacological inhibitors of mitochondrial translation and OXPHOS, we reveal distinct contributions of individual mitochondrial complexes in response to IL-4/IL-13. We also demonstrate a specific, ATP-independent requirement for the mitochondrial ATP synthase in dissipating the mitochondrial membrane potential. We show that ATP synthase–dependent dissipation of the mitochondrial membrane potential prevents membrane hyperpolarization, which activates the integrated stress response (ISR) through the heme-regulated inhibitor (HRI) kinase, ultimately blocking the macrophage response to IL-4/IL-13. Our study extends the current understanding of mitochondrial remodeling in macrophages and establishes the mitochondrial genome as a central determinant of the IL-4/IL-13 macrophage response.

## RESULTS

### Transcript-protein profiling of stimulated macrophages and their mitochondria

**Fig. 1.**
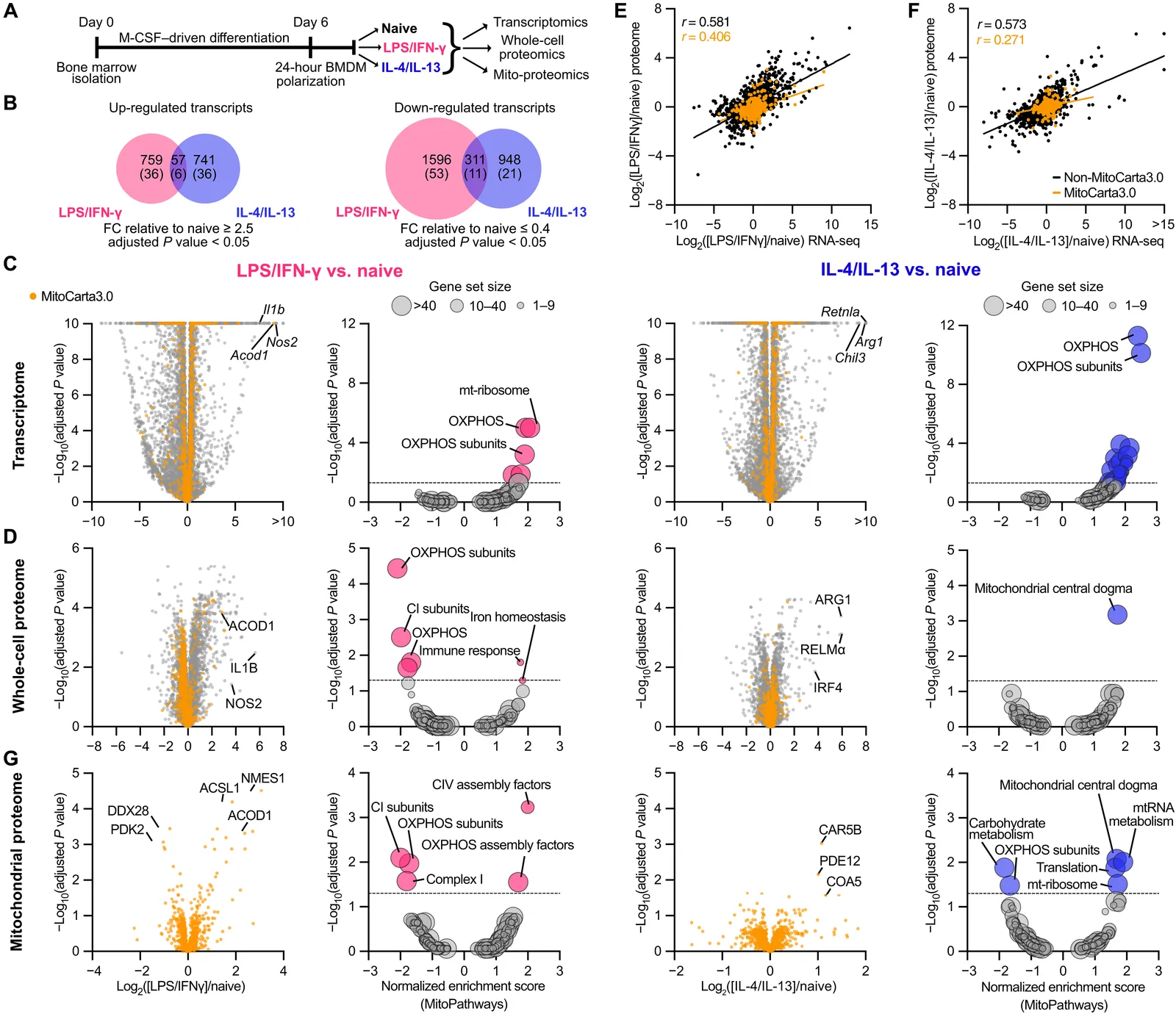
Transcript and protein profiling of polarizing macrophages and their mitochondria. (**A**) Schematic representation of BMDM preparation and treatment. (**B**) Venn diagrams showing significantly up-regulated (left) and down-regulated (right) transcripts following 24 hours of treatment with LPS (100 ng/ml) and IFN-γ (20 ng/mL; pink) or IL-4 (20 ng/mL) and IL-13 (20 ng/mL; blue) relative to naive macrophages at the indicated thresholds. MitoCarta3.0-annotated genes are shown in parentheses. Circle sizes correspond to gene set size. FC, fold change. (**C**) Transcriptome and (**D**) whole cell proteome of isolated mitochondria from 24-hour LPS/IFN-γ– (pink) and IL-4/IL-13– (blue) treated macrophages. Data are expressed relative to naive macrophages (left panels) and GSEA of the indicated datasets using MitoCarta3.0 pathways (right panels). MitoCarta3.0-annotated genes/proteins are highlighted in orange. Significant pathways (adjusted *P* < 0.05) are colored. Circle sizes correspond to gene set size (*N* = 4 mice). (**E** and **F**) Transcript-protein correlation in (F) LPS/IFN-γ–treated and (G) IL-4/IL-13–treated macrophages, covering 7506 and 7479 matched genes/proteins, respectively. Black and orange lines represent trend lines for non-MitoCarta3.0 and MitoCarta3.0 respectively. (**G**) Proteome of isolated mitochondria from 24-hour LPS/IFN-γ– (pink) and IL-4/IL-13– (blue) treated macrophages. Data are expressed relative to naive macrophages (left panels) and GSEA of the indicated datasets using MitoCarta3.0 pathways (right panels). MitoCarta3.0-annotated genes/proteins are highlighted in orange. Significant pathways (adjusted *P* < 0.05) are colored. Circle sizes correspond to gene set size (*N* = 4 mice). DESeq2 was used for the statistical analysis in (C). Student’s *t* test was used for (D) and (E). *P* values were adjusted for multiple comparisons using Benjamini-Hochberg correction test in (C) to (E).

To dissect cellular and mitochondrial remodeling following macrophage stimulation, we generated murine bone marrow–derived macrophages (BMDM) through treatment with macrophage colony-stimulating factor (M-CSF) for 6 days before stimulation with LPS/IFN-γ or IL-4/IL-13 for 24 hours (Fig. 1A). We validated the macrophage response by measuring *Il1b* expression and nitric oxide (NO) production for LPS/IFN-γ treatment and E3 SUMO-protein ligase (*Egr2*) expression and enzymatic arginase activity for IL-4/IL-13 treatment (fig. S1, A to C). We then performed RNA-seq and label-free quantitative proteomics on naive and treated BMDM, quantifying >13,000 transcripts [transcript per million (TPM ≥ 1)] and >8000 proteins (unique peptides ≥ 2) (tables S1 and S2). We found that stimulation substantially affected the transcriptome of macrophages (fig. S1D), affecting 2355 and 1689 transcripts (≥2.5-fold up- or down-regulation, adjusted *P* < 0.05) in LPS/IFN-γ and IL-4/IL-13–treated cells, respectively, a minority of which were shared (Fig. 1, B and C). On the basis of these data, we defined gene sets uniquely up-regulated following LPS/IFN-γ or IL-4/IL-13 treatment, which we then used for downstream analysis as “activation markers” and included canonical up-regulated genes such as *Il1b*, *Nos2*, and *Acod1*/*Irg1* (for LPS/IFN-γ) or *Arg1*, *Retnla*, and *Chil3* (for IL-4/IL-13) (table S3). We then performed the corresponding proteomic analyses, which identified fewer significantly differentially expressed proteins, but showed strong up-regulation of proteins such as ACOD1, IL-1β, and nitric oxide synthase 2 (NOS2) or ARG1, RELMα, and IRF4 in the presence of LPS/IFN-γ and IL-4/IL-13, respectively (Fig. 1D and fig. S1E). On the basis of gene set enrichment analysis (GSEA) using Gene Ontology (GO) terms, we found that, as expected, pathways related to viral infections and IFN responses were the most up-regulated in LPS/IFN-γ–treated cells but were significantly depleted following IL-4/IL-13 treatment, in line with our transcriptome data (fig. S1, F and G) (table S4). In contrast and as shown previously (*36*–*38*), proliferation-related pathways showed a significant increase in IL-4/IL-13–stimulated macrophages.

To characterize mitochondrial remodeling more specifically, we performed GSEA using the list of curated mitochondrial pathways from the MitoCarta3.0 inventory of mitochondrial proteins (table S5) (*39*). Curiously, we found that while OXPHOS was one of the most significantly up-regulated pathways in LPS/IFN-γ–treated cells based on transcript abundance, it was also the most depleted pathway based on protein levels (Fig. 1, C and D), highlighting the value of our combined approach. In contrast, our proteomics analysis of these cells revealed enrichment for pathways related to mitochondrial iron metabolism and immune responses, including elevated ACOD1 levels (Fig. 1D), and a slight decrease (∼10%) in the overall intensity of MitoCarta3.0-mapped peptides (fig. S1H). In IL-4/IL-13–treated macrophages, OXPHOS was also transcriptionally up-regulated, but OXPHOS protein levels and the total abundance of peptides mapping to mitochondrial proteins remained unchanged (Fig. 1, C and D, and fig. S1H). The “mitochondrial central dogma” pathway, encompassing genes involved in mtDNA maintenance and expression, showed an enrichment at the protein level upon IL-4/IL-13 stimulation.

To more systematically analyze these observed discrepancies between transcript and protein abundance, we correlated the expression data for 7506 and 7479 genes detected with high confidence both at the transcript and protein levels in LPS/IFN-γ and IL-4/IL-13–treated macrophages, respectively (Fig. 1, E and F). We observed a positive correlation [Pearson correlation coefficient (*r*) > 0.5] for non-MitoCarta3.0 genes in both LPS/IFN-γ– and IL-4/IL-13–treated macrophages. In contrast, MitoCarta3.0 genes showed lower correlation in both macrophage states (*r* = 0.406 and 0.271, respectively), and no correlation for OXPHOS-related genes in either LPS/IFN-γ– or IL-4/IL-13–treated macrophages (*r* = 0.133 and −0.119, respectively) (fig. S1I), indicating strong posttranscriptional regulation of mitochondrial proteins.

To examine mitochondrial remodeling more directly, we applied our recently developed tag-free protocol to isolate highly pure mitochondria from primary cells (*40*), achieving a 16-fold increase in purity and allowing us to quantify 990 MitoCarta3.0 proteins by mass spectrometry (MS; Fig. 1G and fig. S1, J and K) (tables S2 and S5). Following stimulation, we observed substantial mitochondrial remodeling, particularly in LPS/IFN-γ–induced macrophages (Fig. 1E). Specifically, we confirmed the reduction in OXPHOS protein abundance, especially complex I (CI) subunits, as previously observed (*41*), despite an increase in assembly factors. We also observed increased expression of NMES1, a recently found macrophage-specific complex IV (CIV) subunit (*42*). In IL-4/IL-13–treated cells, we found enrichment of proteins involved in mitochondrial gene expression, mitochondrial RNA (mtRNA) metabolism, and intramitochondrial translation (Fig. 1E), confirming our global proteomics. Collectively, our profiling data provide an extensive resource showing transcriptome and proteome signatures of primary macrophages in response to LPS/IFN-γ or IL-4/IL-13 treatment. Our data highlight the changes underlying mitochondrial adaptation and reveal limited transcript-protein concordance of MitoCarta3.0 genes, especially in IL-4/IL-13–treated cells.

### Mitochondrial genome expression is required for macrophage response to IL-4/IL-13

**Fig. 2.**
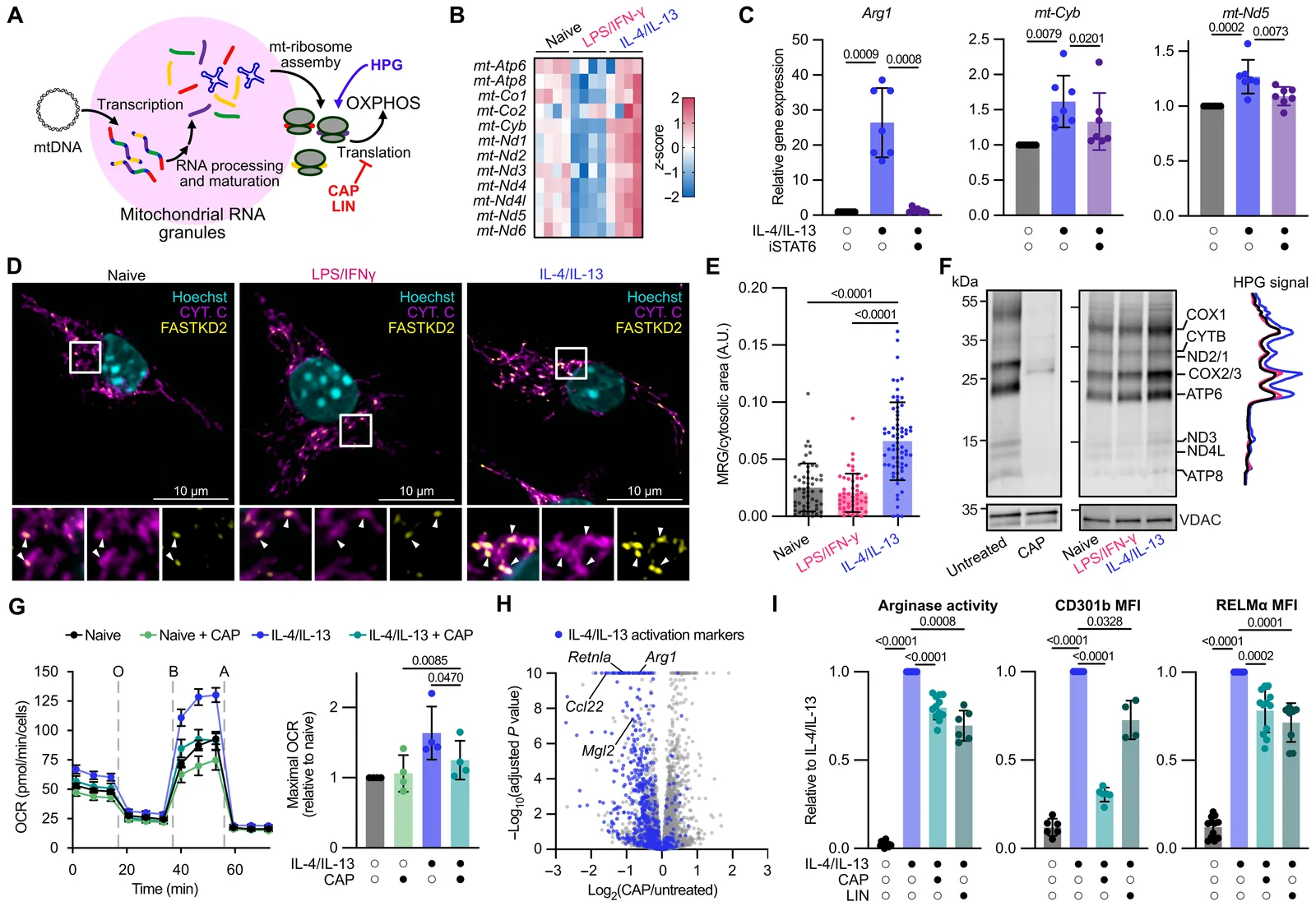
Intramitochondrial protein synthesis supports IL-4/IL-13 macrophage responses. (**A**) Mitochondrial gene expression schematic. (**B**) Heatmap of mtDNA-encoded mRNA levels in naive, LPS/IFN-γ–, and IL-4/IL-13– treated macrophages (*N* = 4 mice). (**C**) qPCR analysis showing transcript levels of *Arg1*, *mt-Cyb*, and *mt-Nd5* in 24-hour IL-4/IL-13–treated BMDM in the presence or absence of the STAT6 inhibitor AS1517499 (iSTAT6), relative to naive (*N* = 7 mice from two independent experiments). (**D**) Representative immunofluorescence of naive, LPS/IFN-γ–, or IL-4/IL-13–treated macrophages labeled for cytochrome c (CYT. C) (mitochondria), mScarlet (FASTKD2), and Hoechst (nuclei). (**E**) MRG abundance quantified from (D) (*N* = 3 mice from three independent experiments). A total of ≥49 cells per condition. A.U., arbitrary units. (**F**) De novo mitochondrial translation visualized by l-homopropargylglycine (HPG) labeling. Twenty-four–hour IL-4/IL-13–treated macrophages with or without the mitochondrial translation inhibitor chloramphenicol (CAP; left); 24 hour–treated macrophages as indicated with their corresponding HPG signal intensity profiles (ImageJ) (right). (**G**) Representative oxygen consumption rate (OCR), and maximal OCR relative to naive in 24-hour IL-4/IL-13–treated macrophages with or without CAP (*N* = 4 mice from four independent experiments). Data are shown as mean ± (left) SEM and (right) SD. O, Oligomycin; B, BAM15; A, Antimycin A. (**H**) Transcriptomics analysis of 24-hour IL-4/IL-13–treated BMDM treated with CAP. IL-4/IL-13 markers are shown in blue (*N* = 4 mice). (**I**) Arginase activity and flow cytometric analysis of CD301b and RELMα levels in IL-4/IL-13 macrophages treated with CAP or linezolid (LIN) relative to naive (*N* ≥ 4 mice from ≥3 independent experiments). MFI, median fluorescence intensity. All data are shown as mean ± SD unless otherwise stated. Paired one-way analysis of variance (ANOVA) with multiple comparisons to untreated IL-4/IL-13 with Dunnett’s correction for (C) and (I); one-way ANOVA with multiple comparisons between all conditions with Tukey’s correction for (E) and (G).

While most mitochondrial proteins are encoded in the nuclear genome, mitochondria also contain their own genome, which encodes a subset of the OXPHOS subunits. Mitochondrial gene expression occurs in the mitochondrial matrix, where transcription initiates on the mtDNA, and nascent RNAs accumulate in mitochondrial RNA granules (MRGs) responsible for processing, maturation, and ribosome assembly (Fig. 2A) (*43*, *44*). Mature ribosomes synthesize the 13 mtDNA-encoded proteins, all of which are core subunits of the OXPHOS complexes. To further our investigation of mitochondrial gene expression following macrophage stimulation, we focused on reads from our RNA-seq data that mapped specifically to the mitochondrial genome. We found that these were significantly decreased in the presence of LPS/IFN-γ and increased in the presence of IL-4/IL-13 (Fig. 2B), consistent with the elevated expression of pathways related to mitochondrial gene expression in IL-4/IL-13 macrophages (Fig. 1, D and E). We validated these differences using reverse transcription quantitative polymerase chain reaction (qPCR) with probes targeting four mtRNAs: *mt-Co1*, *mt-Cyb*, *mt-Nd5*, and *mt-Atp8* (fig. S2A). In parallel, we determined the relative copy number of mtDNA molecules, which we found to be decreased in LPS/IFN-γ but not significantly altered in IL-4/IL-13–treated macrophages (fig. S2B). To investigate the role of the signaling cascade downstream of IL-4/IL-13, we first treated macrophages with AS1517499, a STAT6 phosphorylation inhibitor, and found that the increase in mtDNA-encoded transcripts upon IL-4/IL-13 stimulation depends on STAT6 activity (Fig. 2C). Consistent with the increase in these transcripts, we observed that IL-4/IL-13 treatment also significantly increased MRG abundance, which is tightly linked to mitochondrial transcriptional activity (*21*, *43*, *44*), independently of FASTKD2 protein levels (Fig. 2, D and E, and fig. S2, C and D). On the other hand, we found no difference in total mitochondrial mass following either LPS/IFN-γ or IL-4/IL-13 treatment (fig. S2, B, E, and F), confirming our global proteomics data (fig. S1H).

We extended this analysis by testing intramitochondrial protein synthesis using a clickable methionine analog [l-homopropargylglycine (HPG)] in the presence of the cytosolic translation inhibitor cycloheximide. To validate our assay, we used chloramphenicol (CAP), a clinically used antibiotic that also inhibits mitochondrial ribosome activity, and confirmed that it completely blocked HPG incorporation in IL-4/IL-13–treated macrophages (Fig. 2F). We next measured intramitochondrial protein synthesis in stimulated macrophages and found that while it remained constant in LPS/IFN-γ–treated cells, it significantly increased in the presence of IL-4/IL-13 (Fig. 2F). Together, our observations indicate that mtRNA levels are reduced in LPS/IFN-γ–treated macrophages, whereas IL-4/IL-13 stimulation promotes a STAT6-dependent increase in mtDNA transcription, more abundant MRGs, and increased intramitochondrial protein synthesis, fully consistent with our proteomics analysis (Fig. 1, D and E).

We next tested whether the expression of mtDNA-encoded genes and intramitochondrial translation are necessary for the IL-4/IL-13 response. A hallmark of IL-4/IL-13–mediated stimulation is an increase in mitochondrial respiration relative to naive macrophages (*5*, *6*). Thus, we cotreated cells with IL-4/IL-13 and CAP to test whether this increase relies on de novo mitochondrial protein synthesis. We found that CAP alone did not significantly affect respiration in naive macrophages after 24 hours, as is expected in view of the long half-life of mtDNA-encoded proteins (*45*, *46*). In contrast, CAP effectively blocked the IL-4/IL-13–driven increase in oxygen consumption and maximal respiration (Fig. 2G and fig. S2G), confirming the requirement for de novo intramitochondrial protein synthesis in the characteristic increase in respiration.

To further characterize the effect of inhibiting mitochondrial translation on the IL-4/IL-13 response, we performed RNA-seq of macrophages treated with IL-4/IL-13 in the presence of CAP. We observed a significant decrease in the expression of the downstream IL-4/IL-13 activation markers defined earlier (table S3) (Fig. 2H and fig. S2H), and we confirmed the decreased expression of the canonical markers *Arg1*, *Retnla*, and *Mgl2* by enzymatic assays and flow cytometry (Fig. 2I and fig. S2I). We further validated our findings using the chemically distinct antibiotic LIN, which also targets mitochondrial protein synthesis (Fig. 2I). Neither CAP nor LIN treatment affected macrophage viability (fig. S2J). Together, our data show that the response to IL-4/IL-13, including immunometabolic reprogramming, requires de novo intramitochondrial synthesis of subunits of the mitochondrial respiratory chain.

### Distinct mitochondrial defects block the response to IL-4/IL-13

**Fig. 3.**
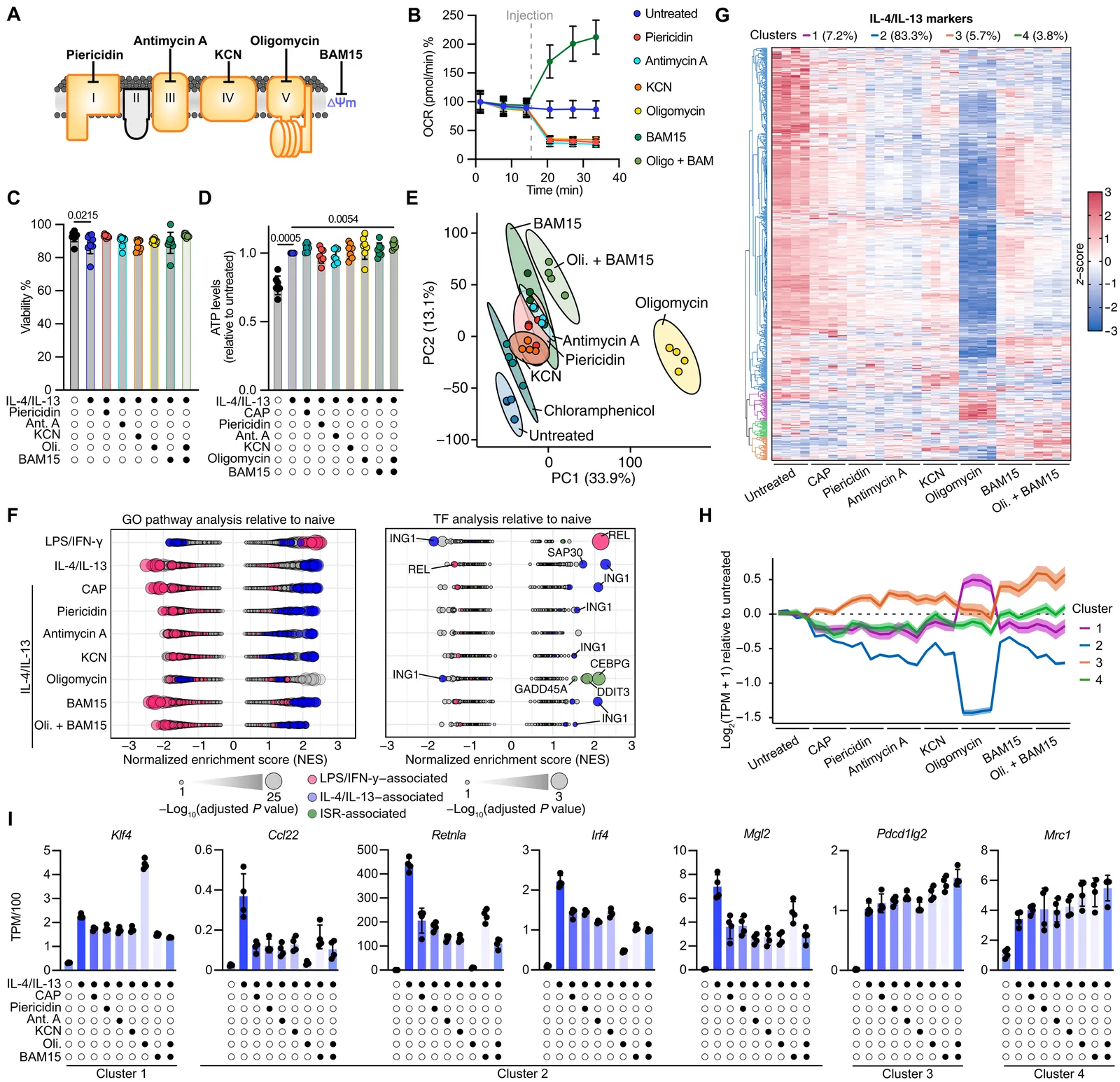
ATP synthase inhibition, rather than OXPHOS, suppresses the response to IL-4/IL-13. (**A**) Schematic of the mitochondrial electron transport chain with mtDNA-encoded complexes in orange and corresponding inhibitors indicated. BAM15 dissipates the mitochondrial membrane potential (ΔΨm). (**B**) Effect of OXPHOS inhibitors on OCR in naive macrophages (*N* ≥ 8 wells, representative of two mice), normalized to the first measurement. (**C**) Viability of IL-4/IL-13–treated macrophages treated for 24 hours with OXPHOS inhibitors (*N* = 6 mice) measured by flow cytometry. Ant. A, Antimycin A; Oli., Oligomycin. (**D**) Relative ATP levels in 24-hour IL-4/IL-13 BMDM treated with different OXPHOS inhibitors (*N* = 7 mice from three independent experiments). (**E**) PCA of RNA-seq profiles from 24-hour IL-4/IL-13 macrophages treated with the indicated inhibitors relative to naive macrophages (*N* = 4 mice). Shaded areas represent 95% data ellipses for each condition. Percentages indicate the total variance explained by each principal component (PC). (**F**) GSEA of pathways (left) and TF signature (right) from (E). Colors indicate association with the responses to LPS/IFN-γ (pink), IL-4/IL-13 (blue), or to the ISR (green). LPS/IFN-γ– or IL-4/IL-13–associated pathways are those with a normalized enrichment score (NES) of ≥2 compared to naive macrophages. Dot sizes correspond to false discovery rate (FDR)–corrected *P* values (adjusted *P* values). (**G**) Unsupervised clustering and heatmap of IL-4/IL-13 marker expression from (E). (**H**) Average expression of genes in each cluster defined in (G). Shaded areas indicate SEM. (**I**) TPM values for representative IL-4/IL-13 markers organized by cluster from (G). All data are shown as mean ± SD unless otherwise stated. Paired one-way ANOVA with multiple comparisons to untreated IL-4/IL-13 with Dunnett’s correction for (C) and (D).

Intramitochondrial protein synthesis is required for the biogenesis of four of the five OXPHOS complexes, whereas only complex II is exclusively nuclear encoded. Having observed a role for mitochondrial translation in the increase in OXPHOS during the IL-4/IL-13 response, we next aimed to test the contribution of each respiratory complex containing mtDNA-encoded subunits to IL-4/IL-13 reprogramming. To this end, we treated BMDM with IL-4/IL-13 in the presence of inhibitors specifically targeting CI (piericidin), III (antimycin A), IV [potassium cyanide (KCN)], or V (oligomycin), as well as with the mitochondrial membrane potential (ΔΨm) uncoupler BAM15, which also suppresses OXPHOS by collapsing the ΔΨm (Fig. 3A). Using respirometry, we confirmed that all reagents reduced mitochondrial respiration to a similar extent, except BAM15, a mitochondria-specific proton ionophore and uncoupler, which increased respiration, as expected (Fig. 3B). None of the drugs affected viability, reduced STAT6 phosphorylation after 24 hours, or lowered ATP levels, as expected from the compensatory increased glycolysis (Fig. 3, C and D, and fig. S3, A and B). Next, we performed RNA-seq on naive, LPS/IFN-γ–, or IL-4/IL-13–treated macrophages, the latter in the absence or presence of CAP or each of the OXPHOS inhibitors. We first analyzed the impact of OXPHOS inhibition in IL-4/IL-13 macrophages using principal components analysis (PCA) and observed that CAP and most OXPHOS inhibitors resulted in a distinct expression profile compared to untreated IL-4/IL-13 macrophages, with oligomycin-treated samples forming a separate cluster from the others (Fig. 3E). Accordingly, when we performed GSEA, using GO terms and TF-based gene sets, we found only mild effects on IL-4/IL-13–associated pathways and TF profiles for all inhibitors, with the notable exception of oligomycin that exhibited a near-complete inhibition of the response to cytokine stimulation (Fig. 3F). We next looked at individual transcripts and performed unsupervised clustering using our list of IL-4/IL-13 markers in these macrophages (Fig. 3, G and H, fig. S3C, and table S1). Our analysis revealed four main clusters of IL-4/IL-13 marker genes. Cluster 1, representing 7.2% of markers, comprised an unexpected set of genes increased only upon oligomycin treatment, including the TF *Klf4* (*47*). Cluster 2, representing 83.3% of markers, was a larger cluster containing most canonical IL-4/IL-13 markers, including *Ccl22*, *Retnla*, *Irf4*, and *Mgl2*. This cluster was affected by all OXPHOS inhibitors, with the strongest effects observed for oligomycin and a milder effect for CAP. Cluster 3, representing 5.7% of markers, comprised a small set of genes increased after BAM15 treatment, including the immune checkpoint ligand *Pdcd1lg2*. Cluster 4, representing 3.8% of markers, contained a small subset of genes with no major effect under any treatment, including *Mrc1* (Fig. 3, G to I, and table S6). We validated these patterns by measuring arginase enzymatic activity, as well as the protein levels of CD206, RELMα, CD301b, and IRF4 (fig. S3, D and E).

We also noted that macrophages treated with a combination of IL-4/IL-13 and oligomycin showed high expression of genes such as *Il6* and *Slc25a33*, which are known as LPS-responsive genes (fig. S3, F to H) (*48*, *49*). However, this group represents less than 5% of the LPS/IFN-γ markers (table S6), of which most were not expressed under these conditions (fig. S3, F and G). Consistently, we found no activation of canonical proinflammatory signaling pathways, such as IκBα), c-Jun N-terminal kinase (JNK), or IRF3 phosphorylation (fig. S3I). Together, our results indicate that all tested OXPHOS inhibitors attenuate mitochondrial respiration and partially suppress IL-4/IL-13–induced macrophage activation. Among these, inhibition of ATP synthase by oligomycin showed the strongest inhibition of the IL-4/IL-13 response but did not engage a proinflammatory program.

### Mitochondrial hyperpolarization induces an HRI-dependent ISR that inhibits the IL-4/IL-13 response independently of OXPHOS

**Fig. 4.**
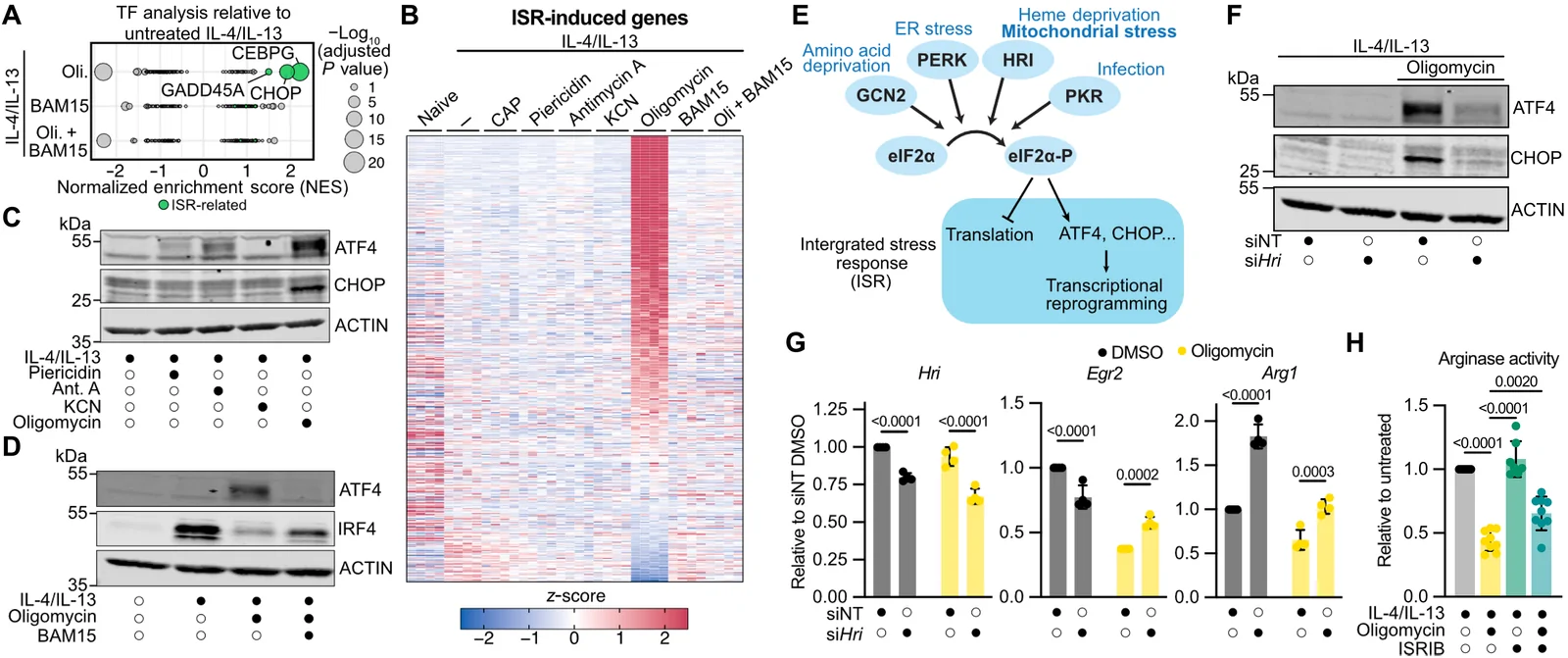
Oligomycin inhibits the response to IL-4/IL-13 through mitochondrial hyperpolarization–dependent activation of the ISR. (**A**) TF GSEA of macrophages cotreated with IL-4/IL-13 and the indicated inhibitors for 24 hours, relative to untreated IL-4/IL-13 macrophages. ISR-related TFs are highlighted in green. Dot sizes correspond to FDR-corrected *P* values (adjusted *P* values). (**B**) Expression levels of ISR-induced genes from transcriptomics analysis of naive or 24-hour IL-4/IL-13–stimulated BMDM treated with OXPHOS inhibitors (*N* = 4 biological replicates). We annotated ISR genes using the list as defined in (*52*). (**C**) Representative immunoblot analysis of ATF4 and CHOP in 24-hour IL-4/IL-13 macrophages treated with OXPHOS inhibitors. (**D**) Representative immunoblot analysis of ATF4, and IRF4 in naive and 24-hour IL-4/IL-13–stimulated BMDM treated with oligomycin in the presence or absence of BAM15. (**E**) Schematic of ISR activation by phosphorylation of the translation factor eIF2α by the four different kinases [GCN2 (general control nonderepressible 2), PERK (protein kinase R–like endoplasmic reticulum kinase), PKR (protein kinase R), and HRI] and their respective activation signals. ER, endoplasmic reticulum. (**F**) Representative immunoblot of ATF4 and CHOP in 24-hour oligomycin-treated IL-4/IL-13 macrophages transfected with small interfering RNA (siRNA) against Hri. (**G**) qPCR analysis of ISR kinase *Hri* and IL-4/IL-13–induced (*Egr2* and *Arg1*) transcripts in 24-hour oligomycin-treated IL-4/IL-13 macrophages transfected with siRNA against Hri (*N* = 4 mice from two independent experiments). DMSO, dimethyl sulfoxide. (**H**) Relative arginase activity (*N* ≥ 7 mice from ≥3 independent experiments) in 24-hour IL-4/IL-13–stimulated BMDM treated with oligomycin and ISRIB, an ISR inhibitor. All data are shown as mean ± SD. Paired two-way ANOVA with multiple comparisons to the respective non-targeting siRNA (siNT) corrected with Šídák test for (G); paired one-way ANOVA with multiple comparisons to oligomycin-treated IL-4/IL-13 corrected with the Dunnett test for (H).

In light of these results, we focused on the unique effects of oligomycin in inhibiting the response to IL-4/IL-13. We noted that in contrast to CAP and the other OXPHOS inhibitors, oligomycin treatment led to a significant increase in the expression of target genes downstream of CHOP, GADD45A, and CEBPG, compared to both naive (Fig. 3F) and IL-4/IL-13 macrophages (Fig. 4A), all of which are related to activation of the ISR (*50*, *51*). This pathway, triggered by both mitochondrial and nonmitochondrial stress, leads to eIF2α phosphorylation and widespread suppression of protein synthesis. Simultaneously, this phosphorylation selectively enhances translation of TFs such as activating transcription factor 4 (ATF4) and CHOP, which induce the expression of downstream stress-responsive genes, including *Asns*, *Shmt2*, and *Ppp1r15a* (encoding GADD34) (*50*, *51*). Consistent with activation of the ISR, we observed a significant and oligomycin-specific increase in the expression of ISR-related genes (*52*), including LPS/IFN-γ markers *Slc25a33* and *Il6*, which in macrophages are reported to be ATF4 targets (Fig. 4B and fig. S3G) (*48*, *53*). We further confirmed a strong accumulation of ATF4 and CHOP/*Ddit3* proteins and transcripts across an oligomycin dose titration, with concentrations as low as 1.6 nM sufficient to replicate the phenotype (Fig. 4C and fig. S4A). We obtained similar results with venturicidin A and N,N′-Dicyclohexylcarbodiimide (DCCD), two chemically distinct ATP synthase inhibitors (fig. S4, B and C), confirming robust ISR activation in response to ATP synthase inhibition.

We next decided to explore the mechanism by which ATP synthase inhibition activates the ISR following IL-4/IL-13 treatment. The primary role of the ATP synthase is to produce ATP; however, we found that ATP levels were maintained following treatment with oligomycin and other OXPHOS inhibitors, indicating that ATP is not the primary driver of the ISR (Fig. 3D). Besides ATP generation, the mitochondrial ATP synthase also plays a crucial and unique role in maintaining the mitochondrial membrane potential (ΔΨm), which is essential for the normal transport of proteins and metabolites across the mitochondrial inner membrane. Accordingly, blocking the ATP synthase results in mitochondrial hyperpolarization, and to test whether this increase in ΔΨm mediates the effects upon ATP synthase inhibition, we cotreated cells with oligomycin, the uncoupler BAM15, and IL-4/IL-13. We found that co-treatment was sufficient to prevent the oligomycin-driven increase in ΔΨm (fig. S4D) and to restore the transcriptional signature following IL-4/IL-13 stimulation, including the IL-4/IL-13 markers *Chil3*, *Irf4*, *Egr2*, or *Arg1* (Fig. 3, E to I, 4D, and fig. S3D). Similarly, BAM15 also prevented the expression of the LPS/IFN-γ markers *Il6* and *Slc25a33* (fig. S3G) and suppressed the ISR transcriptional signature (Fig. 4, A, B, and D). These data strongly suggest that the inhibition of the ATP synthase in IL-4/IL-13–treated macrophages activates the ISR through membrane hyperpolarization. To better understand the temporal sequence between ISR activation and IL-4/IL-13 inhibition, we performed a time-course experiment in oligomycin-treated IL-4/IL-13 macrophages (fig. S4, E and F). At the early time point tested (6 hours), we found a strong induction of ISR transcripts with minimal effect on IL-4/IL-13 markers. In contrast, at later time points, we observed that while the ISR transcripts plateaued, ATF4 protein levels strongly increased, coinciding with the stronger inhibition of IL-4/IL-13 markers.

We next sought to define the mechanism by which mitochondrial membrane hyperpolarization triggers the ISR. Phosphorylation of eIF2α is mediated by four stress-responsive kinases (Fig. 4E) (*54*). We found that their inhibition with small molecules, including heme, failed to prevent ISR activation or to restore IL-4/IL-13 activation markers in oligomycin-treated cells (fig. S5A). However, recent studies have identified a heme-independent role for HRI in relaying mitochondrial stress to the ISR (*55*, *56*). We thus depleted the expression of *Hri* using small interfering RNAs (siRNAs) and observed blunted ATF4 and CHOP induction following oligomycin treatment (Fig. 4F), which was sufficient to partially restore IL-4/IL-13 activation markers, indicating that HRI mediates the ISR in the context of ATP synthase inhibition in macrophages (Fig. 4G).

Multiple insults converge on the ISR, and we next examined whether nonmitochondrial ISR activators would also perturb the macrophage response to IL-4/IL-13. We tested the effect of tunicamycin, an inhibitor of N-linked protein glycosylation in the endoplasmic reticulum (*57*), and Sal003, a salubrinal derivative that inhibits eIF2α dephosphorylation and resolution of the ISR (*58*). We found that both induced the ISR and suppressed the macrophage response to IL-4/IL-13 proportionally to the magnitude of ISR activation, as measured by arginase enzymatic activity, IRF4 protein levels, and IL-4/IL-13 marker gene expression, thus phenocopying the response to oligomycin (fig. S5, B to D, and table S7). Furthermore, we could reduce ISR marker expression and partially restore the response to IL-4/IL-13 in oligomycin-treated macrophages by counteracting the ISR with ISR inhibitor (ISRIB) (Fig. 4H and fig. S5, E and F) (table S7), a compound that overrides the translational block imposed by eIF2α phosphorylation (*59*), or by depleting *Atf4* using siRNAs (fig. S5, G and H). These findings identify the ISR as the mechanistic link between mitochondrial stress and the impaired IL-4/IL-13 response.

**Fig. 5.**
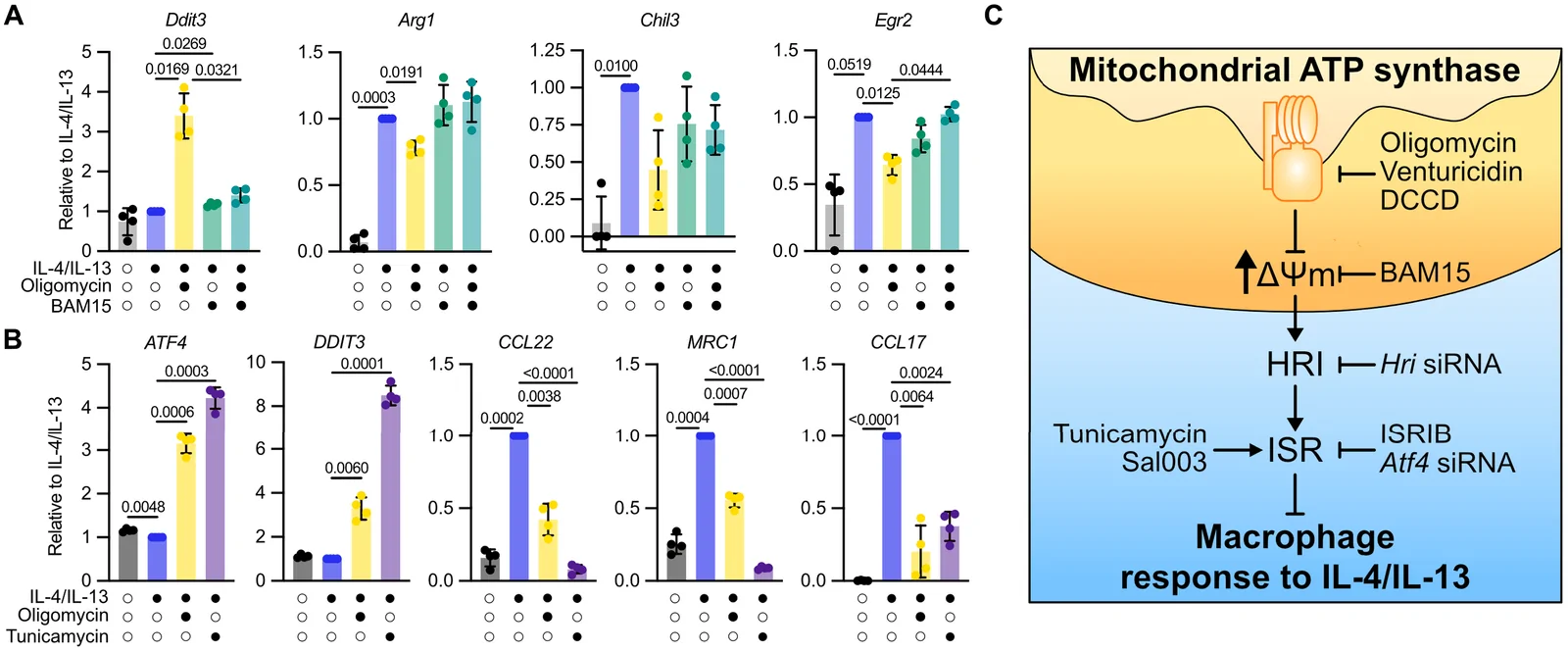
ISR-mediated suppression of IL-4/IL-13 macrophage activation is conserved across macrophage models and species. (**A**) qPCR analysis of *Ddit3*, *Arg1*, *Chil3*, and *Egr2* relative transcript levels in peritoneal macrophages treated for 24-hour with oligomycin and/or BAM15 (*N* = 4 mice from three independent experiments). (**B**) qPCR analysis of the ISR (*ATF4* and *DDIT3*) and IL-4/IL-13 macrophage activation markers (*CCL22*, *MRC1*, and *CCL17*) transcript levels in human monocyte-derived macrophages (hMDMs) treated for 24 hours with oligomycin or tunicamycin (*N* = 4 donors). (**C**) Proposed model: mitochondrial ATP synthase inhibition suppresses the IL-4/IL-13 response in macrophages through membrane hyperpolarization-driven, HRI-dependent activation of the ISR, which can be normalized by BAM15 or ISRIB through dissipation of the ΔΨm or by blocking ISR activation, respectively. Triggering the ISR with tunicamycin or Sal003 also blocks the response to IL-4/IL-13. All data are shown as mean ± SD. Paired one-way ANOVA with multiple comparisons to untreated IL-4/IL-13 and between oligomycin alone and oligomycin/BAM15-treated oligomycin corrected with the Šídák test for (A). Paired one-way ANOVA with multiple comparisons to untreated IL-4/IL-13 corrected with the Dunnett test for (B).

We lastly sought to confirm our findings in additional macrophage models and across species. In mouse peritoneal macrophages, oligomycin induced ISR activation and inhibited IL-4/IL-13 activation, both of which were rescued by BAM15 (Fig. 5A). We observed a similar phenotype in primary human monocyte-derived macrophages isolated from the peripheral blood of healthy donors, where oligomycin and tunicamycin induced the ISR regulators *ATF4* and *DDIT3* (CHOP) and inhibited the expression of IL-4/IL-13 markers *CCL22* and *MRC1* in proportion to ISR activation (Fig. 5B).

Together, our results indicate that mitochondrial ATP synthase inhibition leads to mitochondrial hyperpolarization, which triggers an HRI-dependent ISR and blocks the IL-4/IL-13 response. We show that while alternative ISR triggers also block the IL-4/IL-13 response, the inhibitory effect of the ISR can be reversed using small molecules or siRNAs targeting key nodes in the ISR or by preventing mitochondrial hyperpolarization in the context of mitochondrial ATP synthase inhibition (Fig. 5C).

## DISCUSSION

In this study, we used an integrated approach to characterize the mitochondrial remodeling that occurs in primary macrophages after 24 hours of LPS/IFN-γ or IL-4/IL-13 stimulation, a time point coinciding with the peak of mitochondria-dependent processes such as itaconate production, succinate accumulation, and mtRNA release (*13*, *20*, *30*). Our protein inventory bridges the gap between earlier transcriptional (*12*, *33*), metabolomic (*12*, *30*, *31*), and lipidomics (*60*, *61*) studies, providing an extensive resource. We shared our cellular and mitochondrial proteomics data on the Immunological Proteomic Resource (ImmPRes) platform, an open-access public resource that provides an in-depth, quantitative map of the immunoproteome (https://immpres.co.uk/) (see “Data, code, and materials availability”) (*62*).

We found little or no correlation between transcripts and protein levels for genes encoding mitochondria-localized proteins, including OXPHOS components (Fig. 1, F and G, and fig. S1I). Two reasons likely explain this low correlation: First, in LPS/IFN-γ–treated macrophages, NO promotes degradation of the OXPHOS complexes (*30*, *63*), and second, in IL-4/IL-13–treated macrophages, we observed an acute de novo synthesis of mtDNA-encoded proteins, which are core subunits of OXPHOS complexes and nucleate their assembly. We also showed an increase in the transcriptional activity of TFs associated with proliferation (Fig. 3F), consistent with previous studies (*36*–*38*). In addition, our profiling revealed opposite trajectories for mitochondrial-encoded transcripts following LPS/IFN-γ and IL-4/IL-13 treatment (fig. S2A), suggesting mt-mRNA as a potential biomarker for distinct macrophage states in vivo. In LPS/IFN-γ–treated macrophages, the release of nucleic acids from mitochondria contributes to the immune response and likely explains the decreased abundance of mtRNA we observed after 24 hours (*20*). In contrast, upon IL-4/IL-13 stimulation, we showed that mtRNAs accumulate in a STAT6-dependent manner (Fig. 2C). STAT6 is known to promote expression of TFs and mitochondrial biogenesis factors such as IRF4, PGC-1β, and PPARγ (*6*, *23*, *24*), and it will remain to be determined which factor or combination of factors is responsible for the increase in mtRNA transcription following IL-4/IL-13 treatment. Overall, our inventory demonstrates the need for caution when inferring mitochondrial function and metabolic activity from transcriptomic data alone and highlights the value of quantitative proteomics.

We focused on the immunometabolic remodeling of IL-4/IL-13 macrophages, which leads to a characteristic, yet mechanistically elusive, increase in mitochondrial respiration. Our results indicate that the acute boost in OXPHOS and immune activation of IL-4/IL-13 macrophages requires de novo mtDNA-encoded protein synthesis (Fig. 2, G to I), indicating that mitochondrial gene expression is crucial to the overall macrophage response. Our observations using two chemically distinct antibiotics are consistent with a high-throughput drug screen that identified doxycycline as an inhibitor of IL-4 macrophage activation, both in cell culture and in mouse models of angiogenesis and wound healing. However, this earlier study did not consider a mitochondrial basis for this effect (*64*). Our model is also consistent with results obtained using a macrophage-specific genetic model, which lacks a mitochondrial tRNA synthetase and also exhibits defective OXPHOS and delayed wound healing (*26*). Unlike this genetic model, our pharmacological approach characterizes the contribution of mitochondrial translation without collapsing mitochondrial respiration, distinguishing between these two important mitochondrial functions (Fig. 2G). The milder inhibitory effect of CAP is expected, given the slower kinetics of CAP-mediated OXPHOS inhibition, and highlights how the OXPHOS increase is required for full IL-4/IL-13 responses. Antibiotics that inhibit mitochondrial translation have also been shown to impair the function of immune cells, such as in proinflammatory macrophages or T cells (*34*, *35*, *65*–*67*), a phenotype which was also observed in a clinical study and which could be mitigated by supplementation with uridine and pyruvate (*68*). Previous studies showed that OXPHOS inhibition affects macrophage function (*6*–*8*, *25*), but the extent of this inhibition, the roles of individual respiratory complexes, and their signaling mechanisms were not addressed. Our findings align with these previous studies, and using the present model, we found that antibiotics targeting the mitochondrial ribosome and OXPHOS inhibitors slightly reduced the IL-4/IL-13 response, proportionally to their impact on OXPHOS, but with complex- and marker-specific effects. Together, our observations indicate that the acute increase in OXPHOS following IL-4/IL-13 stimulation is not merely a consequence of the response but also participates in the global reprogramming of these macrophages.

In contrast to previous studies on IL-4/IL-13 macrophages that attributed the effects of oligomycin to reduced OXPHOS (*6*–*8*), we found that ATP synthase inhibition uniquely led to mitochondrial hyperpolarization, triggering an HRI-mediated ISR that suppresses the IL-4/IL-13 response independently of OXPHOS (Fig. 5C). We could reverse this phenotype through both pharmacological and siRNA-mediated inhibition of the ISR. ISRIB had a more limited effect, which is consistent with its reported ability to counteract only mild levels of ISR activation (*69*). Similarly, nonmitochondrial ISR activators also impaired the IL-4/IL-13 response, further supporting the ISR as the central mechanism linking cellular stress to suppression of macrophage activation. Sal003 showed a weaker effect, likely because it inhibits eIF2α dephosphorylation rather than directly inducing eIF2α phosphorylation, and may therefore depend on basal ISR activation levels and concentration. Our results are consistent with recent studies showing ΔΨm-dependent induction of the ISR in nondividing cells (*52*, *70*). While the mechanism linking mitochondrial hyperpolarization to HRI activation in IL-4/IL-13 macrophages remains to be determined, recent findings suggest that membrane potential–dependent OMA1 protease activation may result in cleavage of the mitochondrial messenger protein DELE1 and HRI activation (*55*, *56*). Given the phenotypic diversity of macrophages (*71*–*73*), our results reinforce the need to characterize their state globally rather than through a limited set of markers. Previously, prostaglandin E2 was shown to reduce RELMα levels by a voltage-dependent transcriptional regulation and increase *Arg1* expression (*74*). Consistently, we observed similar results following treatment with BAM15, a mitochondria-specific protonophore. However, we observed a decrease in RELMα with all OXPHOS inhibitors tested, which would indicate regulation by the overall level of OXPHOS rather than by ΔΨm alone, further illustrating the value of comparing multiple inhibitors.

In summary, our study revealed that changes in mitochondrial gene expression and the activity of the ΔΨm-dependent ISR pathway are key determinants in the response of macrophages to IL-4/IL-13. Our findings have direct implications for patients suffering from mitochondrial genetic disorders (*3*, *4*) or undergoing antibiotic treatment because antibiotics that affect mitochondrial gene expression could impair macrophage function. Similarly, the ISR-driven inhibitory mechanism we propose is highly relevant to other medically relevant conditions, such as viral infections or heme or amino acid depletion at sites of infection or in the tumor microenvironment, because these are all well-characterized triggers of the ISR that could block IL-4/IL-13 macrophage function (*75*).

## MATERIALS AND METHODS

### Mice

Wild-type C57BL/6J mice were obtained from Charles River Laboratories. *Fastkd2*-mScarlet-FLAG mice were developed in the Jourdain laboratory and will be reported elsewhere. All animals were bred and housed at two to five per cage in the University of Lausanne (UNIL) Epalinges animal facility. All cages had access to a standard rodent diet and water ad libitum with a 12-hour light/12-hour dark cycle and a stable temperature of 21°C (±1°C) and 55% (±10%) humidity. Age-matched animals of 8 to 13 weeks of age were used for experiments. Unless stated otherwise, all experiments were performed with both males and females. Animals were euthanized using CO_2,_ and all experiments were performed postmortem. All experiments involving animals were conducted under the guidelines of and with approval from the Swiss cantonal veterinary office of Vaud (VD3788).

### BMDM cell culture

Bone marrow was isolated from the femur and tibia, passed through a 40-μm filter, and used for the generation of BMDM in Dulbecco’s modified Eagle’s medium (DMEM)–GlutaMAX (Gibco, 31966021) with 10% heat-inactivated fetal bovine serum (FBS) (Gibco, A5256701), penicillin/streptomycin (100 U/ml; BioConcept, 4-01F00-H) and 10 mM Hepes buffer (BioConcept, 5-31F00-H) supplemented with recombinant murine M-CSF (50 ng/ml; Immunotools, 12343115) on sterile nontreated petri dishes (Thermo Fisher Scientific, 15428784). At day 3 of differentiation, fresh media were supplemented with M-CSF (50 ng/ml). At day 6, BMDM were collected in cold 5 mM EDTA-supplemented phosphate-buffered saline (PBS), counted, and replated at 1 × 10^6^/ml in tissue culture-treated plates for subsequent treatments. BMDM were activated with either LPS (100 ng/ml) O111:B4 (LPS) (Sigma-Aldrich, L4391) and IFN-γ (20 ng/ml; Peprotech, 300-02) or 20 ng/ml of IL-4 (Peprotech, 214-14) and 20 ng/ml of IL-13 (Peprotech, 210-13) for 24 hours unless stated otherwise. Cells were cultured under 5% CO_2_ at 37°C.

When inhibitors were used, BMDM were pretreated for 1 hour with the inhibitor, and then the medium was replaced with medium containing IL-4/IL-13 and the inhibitor for 24 hours unless stated otherwise. The following inhibitors were used: AS1517499 at 5 μM (MedChemExpress, HY-100614), CAP at 100 μg/ml (Sigma-Aldrich, C0378), LIN at 100 μM (MedChemExpress, HY-10394), piericidin at 1 μM (Focus, 10-3467), antimycin A at 1 μM (Sigma-Aldrich, A8674), oligomycin at 1 μM (Tocris, 4110), BAM15 at 10 μM (MedChemExpress, HY-110284), Sal003 at 10 μM (MedChemExpress, HY-15969), tunicamycin at 5 μg/ml (Sigma-Aldrich, T7765), and ISRIB at 1 μM (Sigma-Aldrich, SML0843).

### RNA-seq

RNA extraction was performed on BMDM from four 9-week-old female mice with the RNeasy kit (QIAGEN, 74104) with deoxyribonuclease (DNase) digestion in column ribonuclease (RNase)–free DNase set (QIAGEN, 79254). RNA quality was assessed on a Fragment Analyzer (Agilent Technologies). The RNAs had RNA quality numbers between 8.7 and 9.9. RNA-seq libraries were prepared from 450 ng of total RNA with the Watchmaker mRNA Library Prep Kit (Watchmaker Genomics) using a unique dual indexing strategy and following the official protocol automated on a Sciclone liquid handling robot (PerkinElmer). Libraries were quantified by a fluorometric method (Qubit, Life Technologies), and their quality was assessed on a Fragment Analyzer (Agilent Technologies). Sequencing was performed on an Element Biosciences Aviti with a 2 × 75 cycle sequencing kit Cloudbreak FS High Output (run as single read 150). Base calling and demultiplexing were done with bases2fastq version 2.0 with filter mask R1:N15Y15N*. Purity-filtered reads were trimmed for adapters and quality with Cutadapt (v. 1.8) (*76*). Reads matching to ribosomal RNA sequences were removed with fastq_screen (v. 0.11.1), and remaining reads were further filtered for low complexity with reaper (v. 15-065) (*77*). Reads were aligned against *Mus musculus*.GRCm39.110 genome using STAR (v. 2.7) (*78*). The number of read counts per gene locus was summarized with htseq-count (v. 0.9.1) (*79*). The estimation of the isoforms’ abundance was computed using RSEM (v. 1.2.31) (*80*), and isoform-concatenated transcripts were used to calculate gene-wise TPMs. Quality of the RNA-seq data alignment was assessed using RSeQC (v. 2.3.7) (*81*).

Differential expression analysis was performed on aligned htseq counts using DESeq2 version 1.48.2 (*82*) in R version 4.5.1. *P* values were adjusted using the false discovery rate (FDR) method (Benjamini-Hochberg). Gene set enrichment analyses were performed on DESeq output with clusterProfiler version 4.16.0 (*83*) using mouse GO (M5: GO gene sets BP, CC, and MF) and TF (M3: GTRD gene sets) gene sets obtained from the Molecular Signature Database (*84*) or a curated version of the mouse MitoPathways that included a pathway for the 13 mtDNA-encoded proteins called “mtDNA-encoded” (*39*). Hierarchical clustering of IL-4/IL-13 and LPS/IFN-γ markers was performed on RSEM TPMs [log_2_(TPM + 1)] using hclust() in R, with gene-gene Pearson correlation–based distances (1 – *r*) as input. Correlation heatmaps were visualized using packages dendextend version 1.19.1 (*85*), and ggplot2 version 4.0.0. PCA was performed in R on log_2_(TPM + 1) values, disregarding genes with TPM < 1 in all samples.

### Mitochondria purification and proteomics

BMDM were generated from 16 10-week-old female mice. For each mouse, BMDM were plated in three 15-cm plates at 2 × 10^7^ cells per plate and left stimulated (naive), treated with LPS/IFN-γ or with IL-4/IL-13 in 20 ml of medium as described above. Twenty-four hours after stimulation, plates were washed with PBS, and cells were collected in 10 ml of PBS with a cell scraper. Samples from four different mice were pooled together, generating 12 samples of 40 ml, having three conditions (naive, LPS/IFN-γ, and IL-4/IL-13) from four biological groups. A total of 1.5 ml of each sample was taken and centrifuged at ×300*g* for 3 min, and pellets were kept on ice (whole-cell samples). The rest of the 12 samples were used to isolate mitochondria as described previously (*40*). Whole cell and purified mitochondria were stored at −20°C until protein extraction and MS preparation.

Lysates were digested following a modified version of the in-StageTip (iST) method (*86*) (named miST method). Washed cell pellets were lysed in 100 μl of miST lysis buffer [1% sodium deoxycholate, 100 mM tris (pH 8.6), and 10 mM dithiothreitol] and heated for 10 min at 75°C. After 1:1 (v:v) dilution with water containing 4 mM MgCl^2^ and benzonase (2.5 U/μl; Merck, 70746), samples were incubated for 15 min at room temperature to digest nucleic acids. On the basis of tryptophan fluorescence quantification (*87*), 100 μg of proteins at 1 to 2 μg/μl was transferred to new tubes. Reduced disulfides were alkylated by adding ¼ volume of 160 mM chloroacetamide (32 mM final) and incubating for 45 min at room temperature in the dark. Samples were adjusted to 3 mM EDTA and digested with 1 μg Trypsin/Lys-C mix (Promega, V5073) for 2 hours at 37°C, followed by a second 1-hour digestion with an additional 1 μg of proteases. To remove sodium deoxycholate, two sample volumes of isopropanol containing 1% trifluoroacetic acid (TFA) were added to the digests, and the samples were desalted on a strong cation exchange (SCX) plate (Oasis MCX, Waters Corp., Milford, MA) by centrifugation. After washing with 1% TFA in isopropanol, peptides were eluted in 200 μl of 80% MeCN, 19% water, and 1% (v/v) ammonia and dried by centrifugal evaporation. Samples derived from mitochondrial enrichment (washed pellets) and magnetic-activated cell sorting bead immunoprecipitation were processed similarly to cell pellets but without the benzonase digestion step. For immunoprecipitated samples, digestion was performed on beads for 2 hours with 1 μg of trypsin under gentle agitation. The supernatant was then collected after centrifugation and desalted by SCX solid phase extraction as described above. A dedicated library for data-independent analysis (DIA) was prepared for each type of sample. Aliquots (^1^/_12_) of samples after digestion were pooled (final amount of 12 to 30 μg) and separated into six fractions by offline basic reversed-phase (bRP) using the Pierce High pH Reversed-Phase Peptide Fractionation Kit (Thermo Fisher Scientific). The fractions were collected in 7.5, 10, 12.5, 15, 20, and 50% acetonitrile in 0.1% triethylamine (∼pH 10). Dried bRP fractions were redissolved in 50 to 100 μl of 2% acetonitrile with 0.5% TFA, and 5 to 10 μl was injected for liquid chromatography–tandem mass spectrometry (LC-MS/MS) analyses in data-dependent acquisition (DDA) mode.

LC-MS/MS analyses were carried out on a timsTOF Pro (Bruker, Bremen, Germany) mass spectrometer interfaced through a nanospray ion source (“captive spray”) to an UltiMate 3000 RSLCnano HPLC system (Dionex). Peptides were separated on a reversed-phase custom-packed 45-cm C18 column (75-μm inside diameter, 100 Å; ReproSil-Pur 1.9-μm particles, Dr. Maisch, Germany) at a flow rate of 250 nl/min with a 2 to 27% acetonitrile gradient in 93 min, followed by a ramp to 45% in 15 min and to 90% in 5 min (total method time: 140 min; all solvents contained 0.1% formic acid). Identical LC gradients were used for DDA and DIA measurements. For creation of the spectral library, DDAs were carried out on the six bRP fraction sample pool using a standard thermal ionization mass spectrometry (TIMS) parallel accumulation–serial fragmentation method (*88*) with ion accumulation for 100 ms for each survey MS1 scan and the TIMS-coupled MS2 scans. Duty cycle was kept at 100%. Up to 10 precursors were targeted per TIMS scan. Precursor isolation was done with a 2 thomson (Th) or 3 Th windows below or above mass/charge ratio (*m/z*) of 800, respectively. The minimum threshold intensity for precursor selection was 2500. If the inclusion list allowed it, precursors were targeted more than one time to reach a minimum target total intensity of 20,000. Collision energy was ramped linearly based uniquely on the 1/*k*_0_ values from 20 (at 1/*k*_0_ = 0.6) to 59 eV (at 1/*k*_0_ = 1.6). Total duration of a scan cycle, including one survey and 10 MS2 TIMS scans, was 1.16 s. Precursors could be targeted again in subsequent cycles if their signal increased by a factor of 4.0 or more. After selection in 1 cycle, precursors were excluded from further selection for 60 s. Mass resolution in all MS measurements was approximately 35,000. The data-independent acquisition used mostly the same instrument parameters as the DDA method and was as reported previously (*89*). Per cycle, the mass range of 400 to 1200 *m/z* was covered by a total of 32 windows, each 25 Th wide and a 1/*k*_0_ range of 0.3. Collision energy and resolution settings were the same as in the DDA method. Two windows were acquired per TIMS scan (100 ms) so that the total cycle time was 1.7 s.

Raw Bruker MS data were processed directly with Spectronaut 16.0 (Biognosys, Schlieren, Switzerland). A library was constructed from the DDA bRP fraction data by searching the reference mouse proteome (www.uniprot.org) database of 4 June 2021 (55,364 sequences) and a contaminant database contained as defined in the MaxQuant 16.1.4 software (*90*, *91*). For identification, peptides of 7 to 52 amino acid length were considered, cleaved with trypsin/P specificity and a maximum of two missed cleavages. Carbamidomethylation of cysteine (fixed), methionine oxidation, and N-terminal protein acetylation (variable) were the modifications applied. Mass calibration was dynamic and based on a first database search. The Pulsar engine was used for peptide identification. Protein inference was performed with the IDPicker algorithm. Spectra, peptide, and protein identifications were all filtered at 1% FDR against a decoy database. Specific filtering for library construction removed fragments corresponding to less than three amino acids and fragments outside the 300 to 1800 *m/z* range. In addition, only fragments with a minimum base peak intensity of 5% were kept. Precursors with fewer than three fragments were also eliminated, and only the best six fragments were kept per precursor. No filtering was done on the basis of charge state, and a maximum of two missed cleavages was allowed. Shared (nonproteotypic) peptides were kept. Three libraries were created separately for total cell extracts, enriched, and immunoprecipitated mitochondria but were later combined in a single quantification run with Spectronaut, including all data files. The combined library contained 148,201 unique precursors mapping to 103,792 peptides. Peptide-centric analysis of DIA data was done with Spectronaut 16.0 using the library described above. Single-hit proteins (defined as matched by one stripped sequence only) were kept in the Spectronaut analysis. Peptide quantitation was based on extracted ion chromatogram (XIC) area, for which a minimum of one and a maximum of three (the three best) precursors were considered for each peptide, from which the median value was selected. Quantities for protein groups were derived from interrun peptide ratios based on the MaxLFQ algorithm (*92*). Global normalization of runs/samples was done using the median of peptides. Overall, 147,177 precursors were quantified in the dataset, mapped to 8354 protein groups. Between 6728 and 8167 protein groups were quantified in individual samples (between 67,591 and 92,720 peptides).

All subsequent analyses were done with an in-house developed software tool (available on https://github.com/UNIL-PAF/taram-backend). Contaminant proteins were removed, and quantity values for protein groups generated by Spectronaut were log_2_ transformed. After assignment to groups, only proteins quantified in at least 12 out of 36 samples were kept. Missing values were imputed based on a normal distribution with a width of 0.3 SD, downshifted by 1.8 SD relative to the median. Student’s *t* tests were carried out among conditions, with Benjamini-Hochberg correction for multiple testing (adjusted *P* value threshold < 0.05). Imputed values were later removed.

Before downstream analysis, contaminants introduced during mitochondrial purification, such as immunoglobulins and streptavidin, were removed. For duplicated protein entries, hits annotated as fragments or the least abundant isoform across all samples were excluded. When isoforms showed comparable abundance, both were retained. For the quantification of the enrichment for the different organelles, mitochondrial proteins were annotated with MitoCarta3.0 (*39*), and the other organelles were annotated with the GO database. GSEA was performed with clusterProfiler version 4.16.0 (*83*) using a curated version of the mouse MitoPathways that included a pathway for the 13 mtDNA-encoded proteins called mtDNA encoded. Pearson correlation coefficients between proteomics and RNA-seq data were calculated in R version 4.5.1.

### RNA extraction, reverse transcription, and qPCR

RNA isolation from BMDM was performed with the RNeasy kit (QIAGEN, 74104) or the GenElute Mammalian Total RNA Miniprep Kit (Sigma-Aldrich, RTN350). DNase digestion in column RNase-free DNase set (QIAGEN, 79254) or On-Column DNase I Digestion Set (Sigma-Aldrich, DNASE10), respectively, was used when quantifying mtDNA-encoded transcripts. Reverse transcription was performed with the Moloney murine leukemia virus reverse transcriptase (Promega, M1701) and random primers (Sigma-Aldrich, 11034731001). qPCR was performed with the GoTaq Probe qPCR Master Mix (Promega, A6102) using the following Taqman probes (Thermo Fisher Scientific): Mm00475988_m1 for *Arg1*, Mm00434228_m1 for *Il1b*, Mm00456650_m1 for *Egr2*, Mm00657889_m1 for *Chil3*, Mm01135937_g1 for *Ddit3*, Mm04225271_g1 for *mt-Cyb*, Mm04225315_s1 for *mt-Nd5*, Mm04225236_g1 for *mt-Atp8*, Mm04260177_s1 for *mt-Rnr1*, Mm04225243_g1 for *mt-Co1*, and Mm00438906_m1 for *Tbp*. Plates were acquired with a CFX96 Touch Real-Time PCR system (Bio-Rad) or a LightCycler 480 II (Roche) and acquired for FAM, for our genes of interest, and VIC, for the housekeeping gene TATA-box binding protein (*Tbp*), in each well. All data were normalized to the expression of *Tbp* using the ΔΔ*C*_t_ method.

### mtDNA quantification

RNA-free DNA was isolated using the QIAamp DNA mini kit (QIAGEN, 51304) and RNase A (Machery-Nagel, 740505) following the manufacturer’s instructions. A total of 40 ng of DNA was used for quantification using the GoTaq Probe qPCR Master Mix (Promega, A6102) and the Taqman probes (Thermo Fisher Scientific) Mm04260177_s1 and Mm04225243_g1 for *mt-Rnr1* and *mt-Co1* and Mm00607939_s1 for *Actb* to quantify nuclear DNA. Samples were acquired with a CFX96 Touch Real-Time PCR system (Bio-Rad) or a LightCycler 480 II (Roche). mtDNA probes were acquired using FAM, and *Actb* was acquired using the VIC signal from the same well. Relative mtDNA levels were normalized to *Actb* using the ΔΔ*C*_t_ method.

### Immunofluorescence and microscopy

Cells were fixed in 4% formaldehyde (Thermo Fisher Scientific, 28906) in media at room temperature for 10 min, permeabilized and blocked for 60 min in PBS-0.1% Triton (Sigma-Aldrich, T8787) supplemented with 3% (w/v) bovine serum albumin (BSA) (Sigma Aldrich, A9647) and incubated with anti-mScarlet (15 μg/ml; St John’s Laboratory, STJ140286) and anti-cytochrome c (2.5 μg/ml; BD Biosciences, 556432) for 1 hour at room temperature or at 4°C for 16 hours. Slides were washed three times in PBS–0.1% Triton–3% BSA and incubated with anti-goat immunoglobulin G (IgG) AF-568 (5 μg/ml; Thermo Fisher Scientific, A-11057), anti-mouse IgG AF-488 (Thermo Fisher Scientific, A-21202), and Hoechst (10 μg/ml; Invitrogen, H3570) in PBS–0.1% Triton–3% BSA for 1 hour at room temperature. Coverslips were washed three times in PBS–0.1% Triton–3% BSA and mounted on slides using FluorSave reagent (Merck, 345789). Slides were imaged using an LSM 880 confocal microscope with the Airyscan detector (Zeiss). An automated pipeline on CellProfiler software (https://cellprofiler.org/) was used to quantify MRG by removing the nuclei from the analysis and using a mitochondrial marker for cell delimitation. Live cell images were acquired using a VisiTech iSIM (instant structured illumination microscopy) super-resolution system, built on an Olympus IX83 inverted microscope and equipped with a 100× UPLAPO OHR oil immersion objective (numerical aperture: 1.5) and a Hamamatsu ORCA-Quest CCD camera. All live-cell imaging was performed at 37°C in phenol red–free DMEM FluoroBrite medium. For mitochondrial staining, cells were incubated with 1 μΜ MitoTracker green for 5 min, followed by rinsing with PBS. Mitochondrial volume was quantified using a custom macro in Fiji. Images were background subtracted and Gaussian filtered before mitochondrial masks were generated using combined local (Sauvola) and global (Li) thresholding. The resulting masks were analyzed by three-dimensional segmentation, and mitochondrial volumes were automatically measured. Mitochondrial area was quantified from maximum-intensity *z*-projections using pixel classification in Ilastik, followed by automated image analysis in Python.

### Polyacrylamide gel electrophoresis and immunoblotting

BMDM were washed in PBS and lysed in cold radioimmunoprecipitation assay (RIPA) buffer [25 mM tris (pH 7.5), 150 mM NaCl, 0.1% SDS, 0.1% sodium deoxycholate, 1% NP-40 analog] supplemented with 1/100 protease inhibitor (Sigma-Aldrich, PIC0002) and 1/500 Pierce Universal Nuclease (Thermo Fisher Scientific, 88702). Protein concentration was quantified from lysates using the DC Protein Assay (Bio-Rad, 5000112). Protein extracts were mixed with 5× SDS–polyacrylamide gel electrophoresis (PAGE) loading buffer [250 mM tris (pH 6.8), 4% sodium dodecyl sulfate, 0.06% (w/v) bromophenol blue, 16% (v/v) beta-mercaptoethanol, and 30% (v/v) glycerol]. Gel electrophoresis was done on SDS-PAGE Novex Tris-Glycine Mini Protein gels (Invitrogen, XP08160BOX and XP10200BOX) and transferred to nitrocellulose membranes (Cytiva, 10600004) using a wet transfer chamber with buffer consisting of 0.302% (w/v) tris base, 1.44% (w/v) glycine, and 20% ethanol in water. Membranes were stained with Ponceau S Staining Solution (Thermo Fisher Scientific, A40000278). Immunoblotting was performed after blocking with 5% milk in tris-buffered saline (TBS) [20 mM tris-HCl (pH 7.4) and 150 mM NaCl] with 0.1% Tween 20 (TBS Tween). Primary antibodies were used at 1/500 to 1000 and were as follows: ATF4 (Cell Signaling Technology, 11815), CHOP (Proteintech, 15204-1-AP), IRF4 (Proteintech, 11247-2-AP), mScarlet (St. John’s Laboratory, STJ140286), phospho-STAT6 (Cell Signaling Technology, 9361), STAT6 (BioLegend, 657901), PGC1b (Proteintech, 22378-1-AP), OXPHOS cocktail (Thermo Fisher Scientific, 45-8199), mt-Co1 (Abcam, AB147705-1001), phospho-IKbα (Cell Signaling Technology, 2859S), IKbα (Cell Signaling Technology, 4814), phospho-JNK (Cell Signaling Technology, 4668), JNK (Cell Signaling Technology, 2859), IRF3 (Abclonal, AP0623), ACTIN (Sigma-Aldrich, A3853 and A2066), VDAC (Proteintech, 55259-1-AP). Fluorophore-conjugated secondary antibodies were used at 0.1 μg/ml (LI-COR, 102673-410, 102673-408, 102673-330, and 102673-328). Membranes were imaged by fluorescence detection with Odyssey CLx Imager (LI-COR).

### HPG mitochondrial translation labeling

After 24 hours of stimulation in 15-cm plates, BMDM were washed twice in PBS and then incubated in methionine-free medium (Sigma-Aldrich, D0422) supplemented with 200 μM l-cystine, 4 mM l-glutamine, 1 mM sodium pyruvate, and 10% dialyzed FBS (Sigma-Aldrich, F0392) for 30 min. Cytosolic translation was inhibited by incubating with cycloheximide at 50 μg/ml (Sigma-Aldrich, 01810) for 10 min before the addition of 500 μM HPG (Jena Bioscience, CLK-1067). After 1.5 hours, cells were washed in PBS, collected with a cell scraper, and washed twice in mitochondria isolation buffer (MIB) [10 mM Hepes (pH 7.4), 210 mM mannitol, and 70 mM sucrose]. Mitochondria were isolated as previously described (*40*). Shortly, cell pellets were resuspended in 500 μl of MIB and lysed with two rounds of 25 strokes of a 25-gauge needle. Homogenized suspensions were centrifuged first at ×2000*g* for 5 min at 4°C to remove intact cells and debris, and supernatants were centrifuged at ×3000*g* for 10 min at 4°C. Mitochondrial pellets were resuspended in cold RIPA buffer, and protein quantification was measured as described above. Equal protein amounts were taken from different samples and diluted to the same final volume. One volume of 2× click-reaction solution was added to each sample for a final concentration of 1.2 mM CuSO4 (Jena Bioscience, CLK-MI004), 40 μM picolyl-Azide-PEG4-Biotin (Jena Bioscience, CLK-1167), 10 mM sodium ascorbate (Jena Bioscience, CLK-MI005), 2.4 mM 2-(4-((bis((1-(tert-butyl)-1H-1,2,3-triazol-4-yl)methyl)amino)methyl)-1H-1,2,3-triazol-1-yl) acetic acid (Jena Bioscience, CLK-067), and incubated for 60 min at room temperature. Gel electrophoresis and immunoblot were carried out as described above using 5% BSA–TBS–0.1% Tween as a blocking agent and IRDye 800CW Streptavidin (LI-COR, 926-32230) to detect biotinylated proteins. Membranes were imaged by fluorescence detection with Odyssey CLx Imager (LI-COR). The identification of the different bands was determined by comparison with earlier publications (*93*, *94*).

### Oxygen consumption rate

BMDM were plated in XFe96/XF Pro Cell Culture Microplates (Agilent, 103794-100) at 1 × 10^5^ cells per well. After stimulation, cells were washed twice in PBS, placed in 175 μl of Seahorse XF DMEM (pH 7.4; Agilent, 103575-100) supplemented with 25 mM glucose (Sigma-Aldrich, G7021), 2 mM l-glutamine (BioConcept, 5-10K50-H), and 1 mM pyruvate (Gibco, 11360070) and incubated for 1 hour at 37°C without CO_2_. Oxygen consumption rate (OCR) was measured by a Seahorse XFe96 Analyzer (Agilent) at the basal state and after being sequentially perturbed with 1 μM oligomycin A (Tocris, 4110), 10 μM BAM15 (MedChemExpress, HY-110284), and 1.6 μM antimycin A (Sigma-Aldrich, A8674). Data were analyzed with Seahorse Wave Desktop software (Agilent). OCR was normalized by cell count obtained by nuclei staining with Hoechst (10 μg/ml; Invitrogen, H3570) after fixation with 4% formaldehyde (Thermo Fisher Scientific, 28906) in PBS for 10 min at room temperature. Images were acquired with an EVOS M7000 (Thermo Fisher Scientific), and automated nuclei counting was done with the EVOS analysis software (Thermo Fisher Scientific, version 1.6.2563.149). Spare respiratory capacity was calculated by subtracting average basal OCR from maximal OCR.

### Arginase assay

Arginase assay was performed using the Arginase Activity Assay Kit (Sigma-Aldrich, MAK112) and following the manufacturer’s instructions. In short, 1 × 10^6^ BMDM were collected in 200 μl of RIPA-Arg buffer [10 mM tris (pH 7.4) and 0.4% (v/v) Triton X-100], protein concentration was quantified, and 0.5 to 1 μg of each sample was transferred in duplicates, sample and blank well, in a 96-well plate for the arginase assay. Volume was brought to 40 μl with dH_2_O, and 10 μl of substrate buffer was added to each sample well before incubating the plate for 2 hours at 37°C. To stop the arginase reaction, urea reagent was added to all wells, and 10 μl of substrate buffer was added to the blank wells before measuring absorbance at 430 nm with a BioTek Synergy H1 microplate reader (Agilent). Arginase activity was normalized to protein concentration.

### Flow cytometry

BMDM were stained with Zombie fixable viability NIR (BioLegend, 423105) or Violet (BioLegend, 423113) in PBS for 20 min at 4°C and washed in PBS. Cell surface staining was performed in fluorescence-activated cell sorting (FACS) buffer (PBS supplemented with 2 mM EDTA and 5% FBS) for 60 min at 4°C with PE anti-mouse CD301b (2 μg/ml; BioLegend, 146803) and APC anti-mouse CD206 (2 μg/ml; BioLegend, 141707). For intracellular staining, cells were fixed and permeabilized with the Foxp3 TF staining buffer set (Thermo Fisher Scientific, 00-5523-00) following the manufacturer’s protocol and stained with anti-murine RELMα (5 μg/ml; Peprotech, 500-P214) and anti-rabbit IgG AF-488 (5 μg/ml; Thermo Fisher Scientific, SAB4600234). Before acquisition, all samples were washed in FACS buffer. Samples were acquired on a CytoFLEX S (Beckman Coulter) and analyzed with FlowJo v10.

### Griess reaction

Griess assay was performed using the Griess Reagent System (Promega, G2930) and following the manufacturer’s instructions. In short, BMDM plated in a 96-well plate were centrifuged at ×300*g* for 3 min, and 60 μl of supernatant was collected and stored on ice. BMDM were washed in PBS, and 25 μl of cold RIPA buffer was added to each well to quantify protein concentration. A total of 50 μl of supernatant was transferred to a 96-well plate for the Griess reaction. The assay was performed by adding 50 μl of sulfanilamide solution and 10 min of incubation at room temperature, followed by adding 50 μl of NED solution per well. Absorbance was measured at 540 nm with a BioTek Synergy H1 microplate reader (Agilent). Nitrite concentrations were calculated using a standard and normalized by the respective protein concentration in each well.

### ATP level quantification

BMDM were plated in 96-well plates at 1 × 10^5^ cells per well. After stimulation, ATP was quantified using the CellTiter-Glo luminescent cell viability assay kit (Promega, G9242) according to the manufacturer’s instructions. Briefly, 100 μl of reconstituted CellTiter-Glo Reagent was added to the wells and mixed by orbital shaking for 2 min. After a 10-min incubation at room temperature to stabilize the luminescence signal, the well content was transferred to an opaque-walled 96-well plate. Data were acquired with a BioTek Synergy H1 microplate reader (Agilent).

### Mitochondrial membrane potential measurement

BMDM were plated in 12-well plates at 1 × 10^6^ cells per well. After stimulation, cells were incubated with 10 nM MitoTracker Red CMXRos (Thermo Fisher Scientific, M7512) in the presence of the different inhibitors for 30 min at 37°C in medium. After aspirating the medium, cells were collected in cold 5 mM EDTA-supplemented PBS, washed with FACS buffer, and acquired on a CytoFLEX S (Beckman Coulter) and analyzed with FlowJo v10.

### siRNA-mediated knockdown in BMDM

siRNA-mediated knockdowns were performed in BMDM differentiated as described above. Reverse transfections were carried out using Lipofectamine RNAiMAX Transfection Reagent (Thermo Fisher Scientific, 13778030) in BMDM medium supplemented with M-CSF. Cells were plated at 0.4 × 10^6^ cells per well in 24-well plates and transfected with siPOOLs (siTOOLS Biotech) at a final concentration of 25 nM. The following siPOOLs were used: si-Eif2ak1/Hri (si-G020-15467) and si-Atf4 (si-G020-11911). A nontargeting control siPOOL (si-C002) was used as a negative control.

For *Atf4* knockdowns, cells were maintained in the transfection mixture for 16 hours before stimulation with IL-4/IL-13, with or without oligomycin, using the same conditions described for the other experiments. For *Hri* knockdown experiments, cells were maintained in the transfection mixture for 16 hours, followed by an additional 24-hour recovery period in fresh BMDM medium supplemented with M-CSF, to achieve maximal RNA and protein degradation before stimulation. Knockdown efficiency was validated by qPCR for *Hri* and by qPCR and immunoblotting for *Atf4*, as outlined above.

### Peritoneal macrophages

Using a 26-gauge needle, 3 ml of ice-cold PBS supplemented with 5 mM EDTA was injected into the exposed peritoneal cavity, followed by gentle massage of the abdominal wall for 20 to 30 s. The peritoneal lavage was collected and pooled with the lavage from three to four additional mice. After centrifugation at ×300*g* for 3 min, the cell pellet was resuspended in 10 ml of DMEM-GlutaMAX (Gibco, 31966021) supplemented with 10% heat-inactivated FBS, penicillin/streptomycin (100 U/ml), and 10 mM Hepes buffer and counted. Peritoneal lavage cell suspension was plated in 24-well plates at 1 × 10^6^ cells per well. After 3 to 4 hours, cells were washed twice with PBS to remove nonadherent cells and treated as described above for BMDM. After 24 hours of stimulation, RNA was extracted with TRIzol (Thermo Fisher Scientific, 15596026). Reverse transcription and qPCR were performed as described above.

### Human monocyte-derived macrophages

Human blood samples from healthy donors were obtained from a local transfusion center (Centre de transfusion sanguine in Lausanne-Epalinges, 1066 Epalinges, Switzerland). Samples were collected and provided by the Interregional Blood Transfusion SRC (Bern, Switzerland), which obtained informed consent from all donors for research use of their samples. The use of human blood was approved under project number P_389. Peripheral blood mononuclear cells were isolated by density-gradient centrifugation using Lymphoprep (STEMCELL, 07851). CD14^+^ monocytes were purified using CD14 microbeads (Miltenyi, 130050201) according to the manufacturer’s instructions and were plated in RPMI 1640 (Gibco, 61870010) supplemented with recombinant human M-CSF (50 ng/ml; ImmunoTools, 11343113), 10% heat-inactivated FBS, penicillin/streptomycin (100 U/ml), and 10 mM Hepes. After 3 days, the medium was replenished with M-CSF–supplemented RPMI 1640. On day 6, cells were detached, counted, and replated at 0.8 × 10^6^ cells per well of a 12-well plate. Polarization and inhibitor treatments were performed using the same conditions as for BMDM.
