# Supplementary material for: Mitochondrial profiling across macrophage states reveals inhibition of IL-4/IL-13 reprogramming by the integrated stress response
Source: Sci Adv. 2026 Aug 19;12(34):eaed6318. doi: 10.1126/sciadv.aed6318 (PMC13488931; doi:10.1126/sciadv.aed6318)
Supplement: Supplementary file 1 [file sciadv.aed6318_sm.pdf]

Science Advances
AAAS

Supplementary Materials for

Mitochondrial profiling across macrophage states reveals inhibition of IL-4/ IL-13 reprogramming by the integrated stress response

Joan Blanco-Fernandez *et al.*

Corresponding author: Alexis A. Jourdain, alexis.jourdain@unil.ch

Sci. Adv. **12**, eaed6318 (2026)
DOI: 10.1126/sciadv.aed6318

The PDF file includes:

Figs. S1 to S5
Legends for tables S1 to S7

Other Supplementary Material for this manuscript includes the following:

Tables S1 to S7

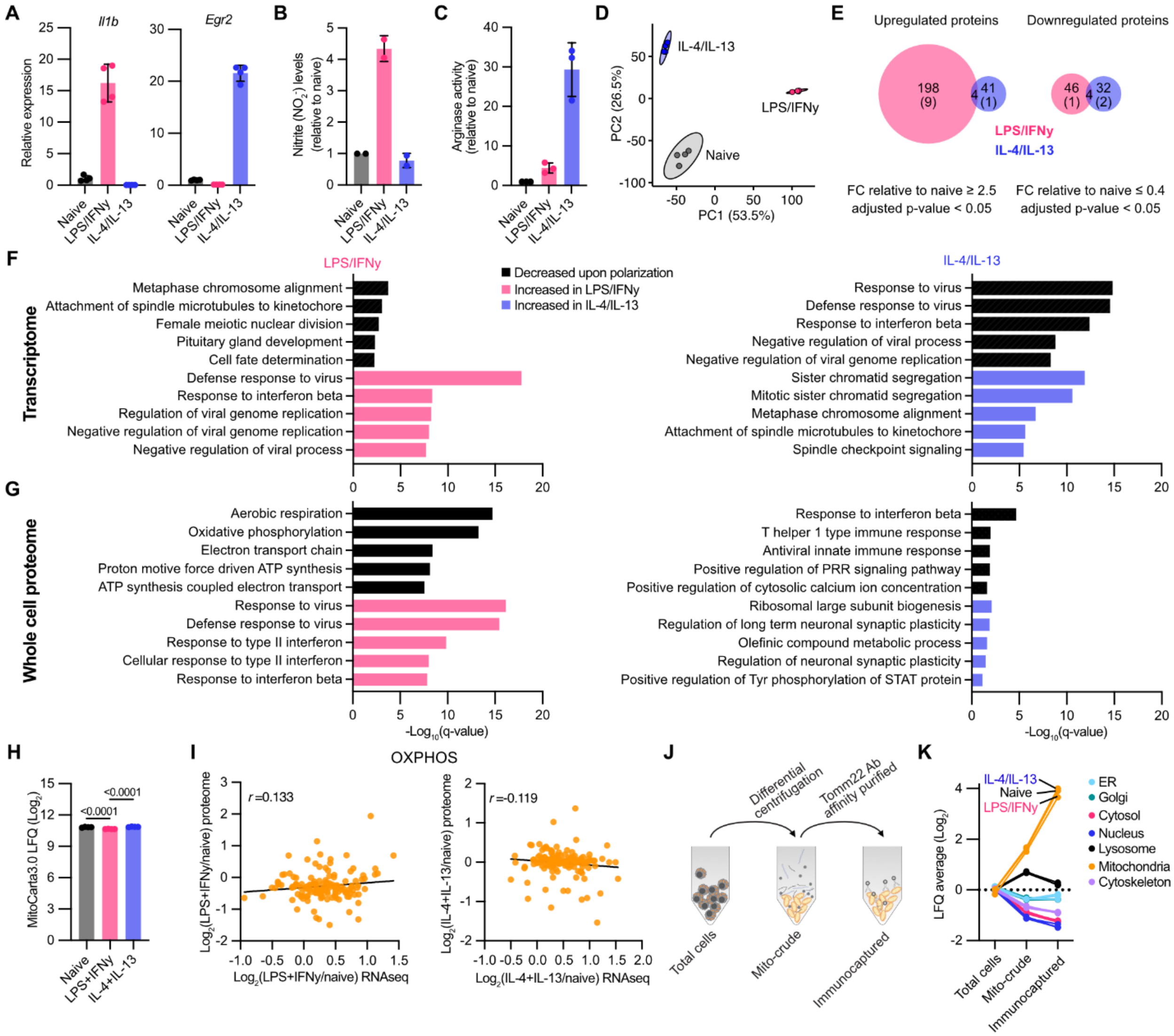

Supplemental Figure 1. Validation and extended analysis of transcriptomic and proteomic profiling of stimulated macrophages and their mitochondria. (A) qPCR analysis of *Il1b* and *Egr2* relative transcript levels in 24 h-treated BMDM (N = 4 mice from a representative experiment). **(B)** Relative levels of nitrite in the supernatant of 24 h-treated BMDM (N = 2 mice from a representative experiment). **(C)** Relative arginase activity in 24 h-treated BMDM (N = 3 mice from a representative experiment). **(D)** Principal component analysis (PCA) of RNA-seq profiles from 24 h naive, LPS/IFNγ, and IL-4/IL-13 macrophages (N = 4 mice). Shaded areas denote variance of each condition. Percentages represent the proportion of total variance captured by each principal component (PC). **(E)** Venn diagrams of significantly upregulated (left) and downregulated (right) proteins following 24 h LPS/IFNγ (pink) and IL-4/IL-13 (blue) treatment relative to naive macrophages at the indicated thresholds. MitoCarta3.0-annotated genes are in parentheses. **(F, G)** The 5 most significantly upregulated and downregulated pathways from a Gene Ontology-based GSEA following treatment with LPS/IFNγ (left) and IL-4/IL-13 (right) treatment. Data extracted from transcriptomics (F) and whole cell proteomics (G) are expressed relative to naive macrophages. **(H)** Label-free quantification (LFQ) showing averaged levels of MitoCarta3.0-annotated proteins from naive and 24 h-treated LPS/IFNγ and IL-4/IL-13 macrophages (N = 4 biological replicates). **(I)** Transcript-protein correlation in macrophages

treated for 24 h with LPS/IFNγ- (left) and IL-4/IL-13 (right), showing matched genes/proteins corresponding to the MitoCarta3.0-defined "OXPHOS" pathways. r: Pearson correlation. **(J)** Scheme of the tag-free mitochondrial isolation protocol. **(K)** LFQ showing average levels of proteins from seven organelles derived from naive and 24 h-treated macrophages using the tag-free mitochondria isolation protocol (N = 4 biological replicates). All data are shown as mean ± SD. One-way ANOVA with multiple comparisons corrected with the Tukey test for (H)**.**

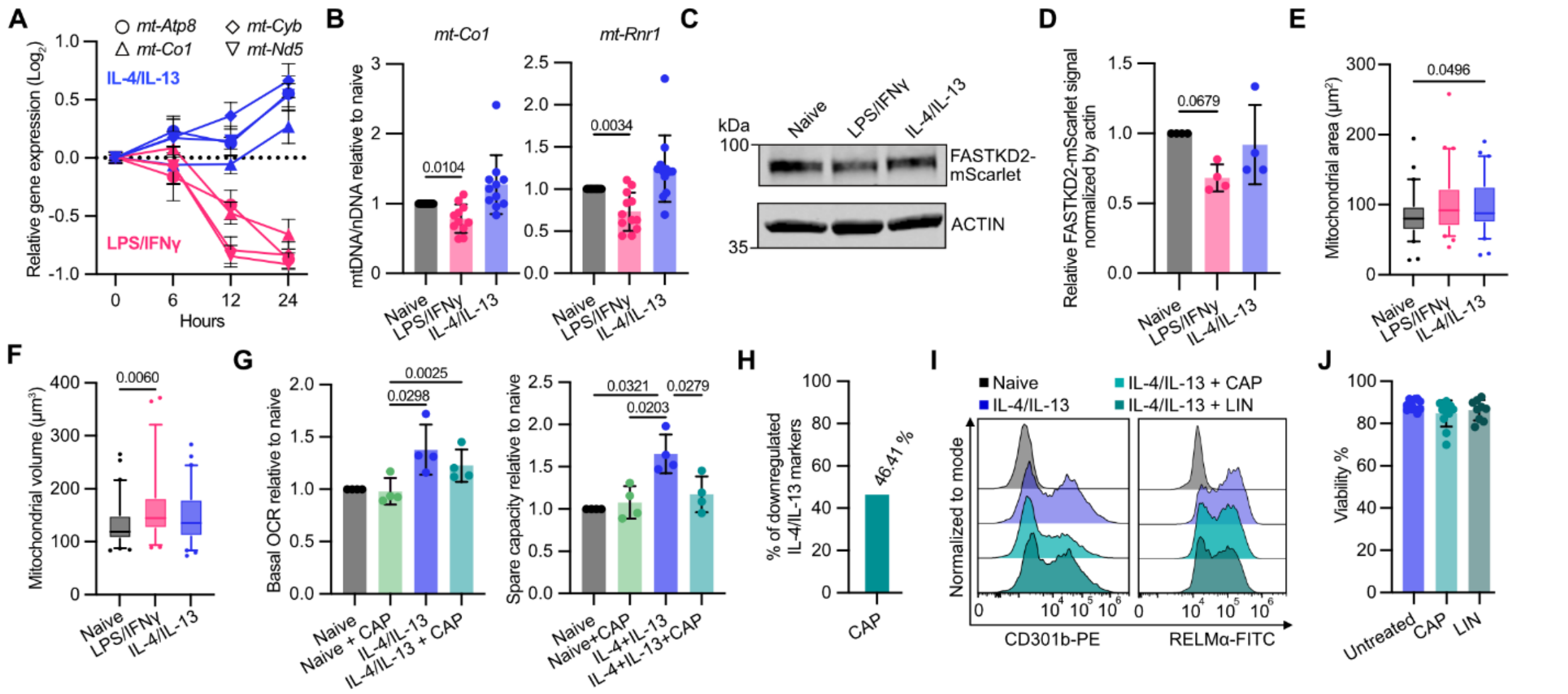

Supplemental Figure 2. Extended analysis of mitochondrial gene expression and mitochondrial translation inhibition in IL-4/IL-13 macrophages. (A) Time point qPCR of *mt-Atp8*, *mt-Co1*, *mt-Cyb*, and *mt-Nd5* in LPS/IFNγ- or IL-4/IL-13-treated BMDM relative to 0 h (naive). Each dot represents the average of ≥ 8 mice from 3 independent experiments. **(B)** mtDNA abundance in BMDM treated for 24 h with LPS/IFNγ or IL-4/IL-13 relative to naive BMDM (N = 11 mice from 4 independent experiments). nDNA: nuclear DNA. **(C)** Representative immunoblot of FASTKD2-mScarlet in naive, and in 24 h-treated LPS/IFNγ, or IL-4/IL-13 BMDM. **(D)** FASTKD2-mScarlet quantification (N = 4 mice from 2 independent experiments) of (C). **(E, F)** Live imaging quantification of (E) mitochondrial area and (F) volume in 24 h LPS/IFNγ and IL-4/IL-13-stimulated macrophages. (N = 3 mice from 3 independent experiments). ≥ 49 cells quantified in each condition. Whiskers are 5-95 percentile. **(G)** Relative basal oxygen consumption rate (OCR) (left) and spare respiratory capacity (right) in naive and 24 h IL-4/IL-13-treated macrophages in the presence or absence of chloramphenicol (CAP) (N = 4 mice from 4 independent experiments). **(H)** Percentage of the significantly downregulated (FDR-adjusted p-value < 0.05) IL-4/IL-13 markers out of 627 in 24 h IL-4/IL-13-stimulated macrophages treated with chloramphenicol (CAP). **(I)** Representative histograms of (left) surface CD301b-PE and (right) intracellular RELMα-FITC fluorescence intensity in naive or 24 h stimulated BMDM with IL-4/IL-13 treated with CAP or linezolid (LIN). **(J)** Viability of naive and 24 h-treated BMDM with IL-4/IL-13 in the presence of CAP and LIN. All data are shown as mean ± SD unless otherwise stated. Paired one-way ANOVA with multiple comparisons between all conditions corrected with the Tukey test for (D) and (G). Paired one-way ANOVA with multiple comparisons to naive or untreated IL-4/IL-13 corrected with the Dunnett test for (B) and (J) respectively. Non-parametric one-way ANOVA with multiple comparisons between all conditions corrected with Dunnett's test for (E) and (F). Only p-values < 0.05 are shown, except for (D).

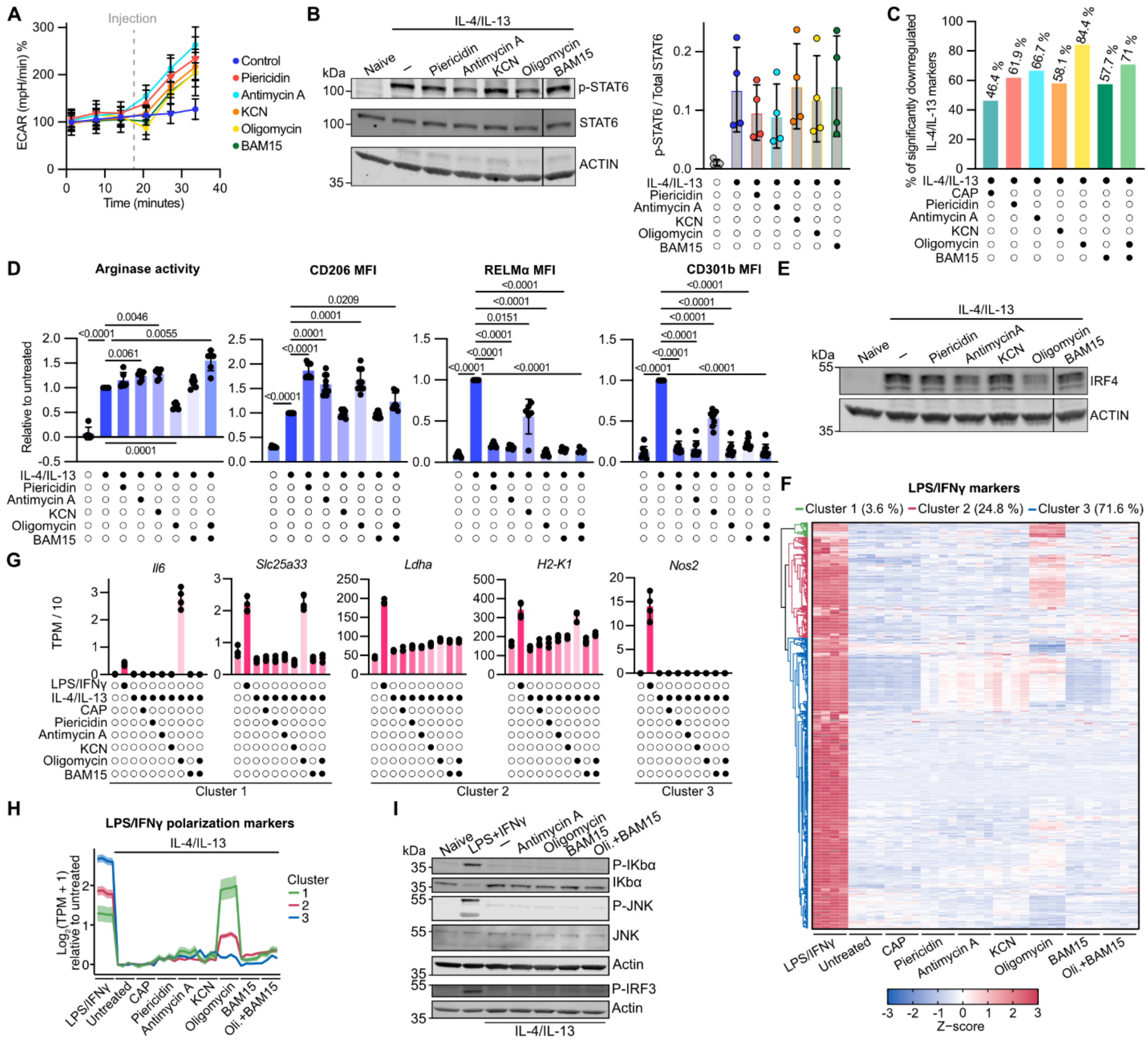

Supplemental Figure 3. OXPHOS inhibition diminishes the IL-4/IL-13 response in macrophages without inducing the LPS/IFNγ phenotype. (A) Effect of OXPHOS inhibitors on the extracellular acidification rate (ECAR) in naive macrophages. (N ≥ 8 well replicates, representative of 2 mice), normalized to the first measurement. KCN: Potassium cyanide. **(B)** (Left) Representative immunoblot showing total STAT6 and its phosphorylation on Tyr641 in naive and 24 h IL-4/IL-13-stimulated BMDM treated with OXPHOS inhibitors; (right) pSTAT6 quantification (N = 4 mice). **(C)** Percentage of significantly downregulated (FDR-adjusted p-value < 0.05) IL-4/IL-13 markers of the 627 marker proteins in 24 h IL-4/IL-13-stimulated BMDM following treatment with the indicated inhibitors. CAP: Chloramphenicol. **(D)** Arginase activity and relative levels of CD206 (surface), RELMα (intracellular) and CD301b (surface), as determined by immunolabeling and flow cytometry in 24 h IL-4/IL-13-stimulated BMDM treated with the indicated OXPHOS inhibitors. (N ≥ 6 mice from ≥ 2 independent experiments). MFI: median fluorescence intensity. **(E)** Representative immunoblot of IRF4 in naive and 24 h stimulated IL-4/IL-13 BMDM treated with OXPHOS inhibitors. Vertical line: lane splicing of the same gel. **(F)** Unsupervised clustered analysis and heatmap of the expression levels of LPS/IFNγ markers present in the RNA-seq profiles of 24 h IL-4/IL-13 macrophages treated with the indicated

inhibitors, and relative to naive macrophages (N = 4 mice). Oli.: Oligomycin. **(G)** Transcript per million (TPM) values for representative LPS/IFNγ markers from (F). **(H)** Averaged expression of the genes in each cluster defined in (F). Shaded areas along each trace represent SEM. **(I)** Representative immunoblot analysis of phosphorylated and total IκBα (nuclear factor of kappa light polypeptide gene enhancer in b-cells inhibitor alpha), JNK (c-Jun N-terminal kinase) and phosphorylated IRF3 (interferon regulatory factor 3) in naive, 24 h LPS/IFNγ-BMDM, and IL-4/IL-13-stimulated BMDM treated with the indicated OXPHOS inhibitors. All data are shown as mean ± SD unless otherwise stated. Paired one-way ANOVA with multiple comparisons to untreated IL-4/IL-13 corrected with the Dunnett test for (B). One-way ANOVA with multiple comparisons between all conditions corrected with the Tukey test for (D)**.** Only p-values < 0.05 are shown.

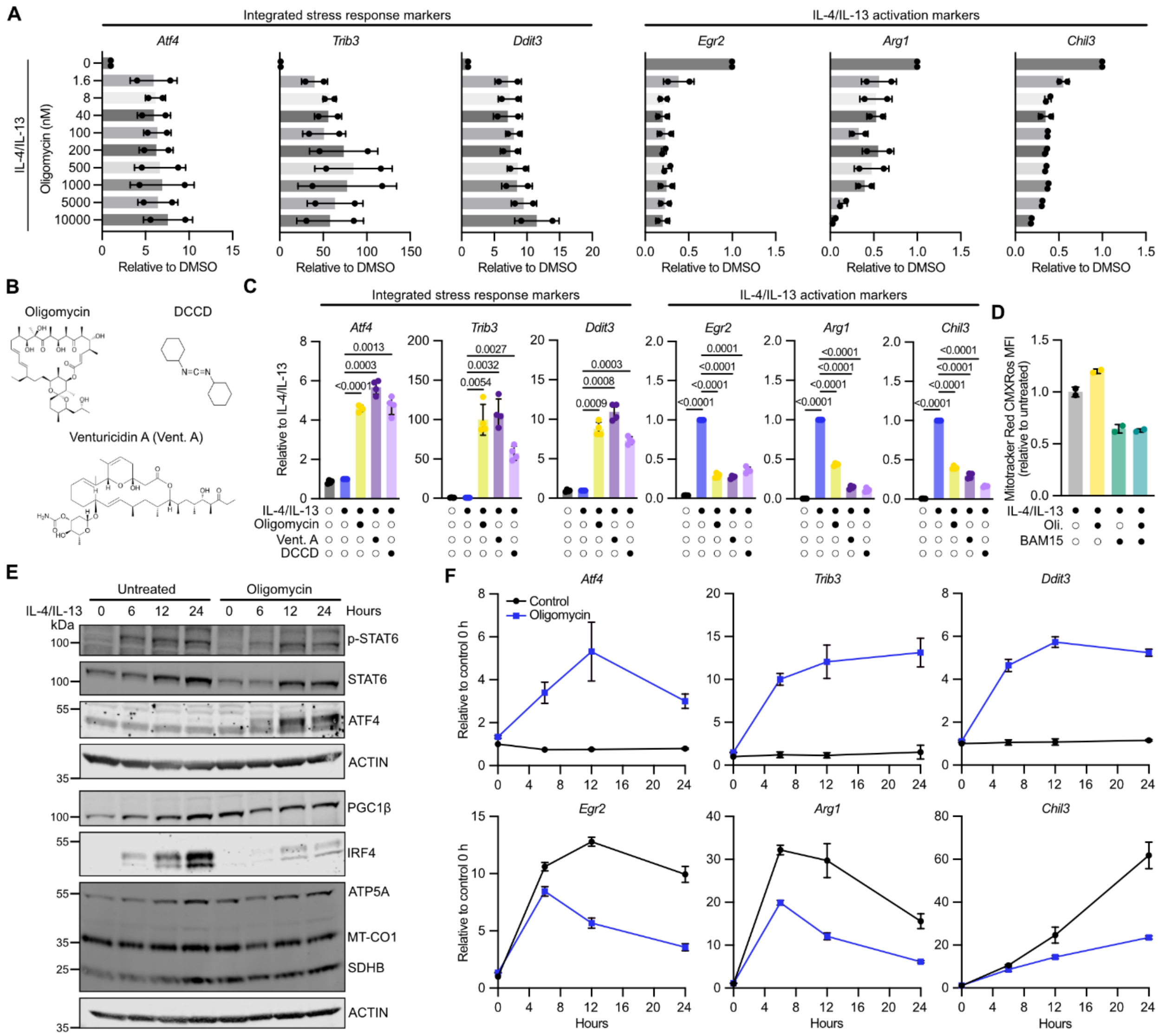

Supplemental Figure 4. ATP synthase inhibition induces a sustained integrated stress response (ISR) that suppresses IL-4/IL-13 macrophage activation. (A) Dose-response qPCR analysis of ISR-induced genes (*Atf4*, *Trib3*, and *Ddit3*) and IL-4/IL-13 markers (*Egr2*, *Arg1*, and *Chil3*) transcript levels in 24 h IL-4/IL-13-stimulated macrophages treated with oligomycin (N = 2 mice from a single experiment). **(B)** Chemical structure of ATP synthase inhibitors oligomycin (left), venturicidin A (Vent.A, bottom), and N,N′-Dicyclohexylcarbodiimide (DCCD, right). **(C)** qPCR analysis of ISR-induced genes (*Atf4*, *Trib3*, and *Ddit3*) and IL-4/IL-13 markers (*Egr2*, *Arg1*, and *Chil3*) transcript levels in 24 h IL-4/IL-13-stimulated macrophages treated with ATP synthase inhibitors shown in (B) (N = 4 mice from two independent experiments). **(D)** Flow cytometry quantification of MitoTracker Red CMXRos signal in IL-4/IL-13 BMDM treated with oligomycin (Oli.), BAM15, or oligomycin with BAM15 for 1 h. MFI: median fluorescence intensity. (N = 2 technical replicates from one mouse). **(E)** Representative immunoblot analysis of total STAT6, p-Tyr641 STAT6, ATF4, PGC1β, IRF4 and OXPHOS subunits ATP5A (complex V), MT-CO1 (complex IV), and SDHB (complex II) in IL-4/IL-13 macrophages treated with oligomycin at the indicated time points. **(F)** Relative gene expression levels of (top) ISR-induced genes (*Atf4*, *Trib3*, and *Ddit3*) and (bottom) IL-4/IL-13 activation markers (*Egr2*, *Arg1*, and *Chil3*) in 0, 6, 12, 24 h

IL-4/IL-13 macrophages treated with oligomycin (N = 3 mice). All data are shown as mean ± SD. Paired one-way ANOVA with multiple comparisons to untreated IL-4/IL-13 corrected with the Dunnett test for (C). Only p-values < 0.05 are shown.

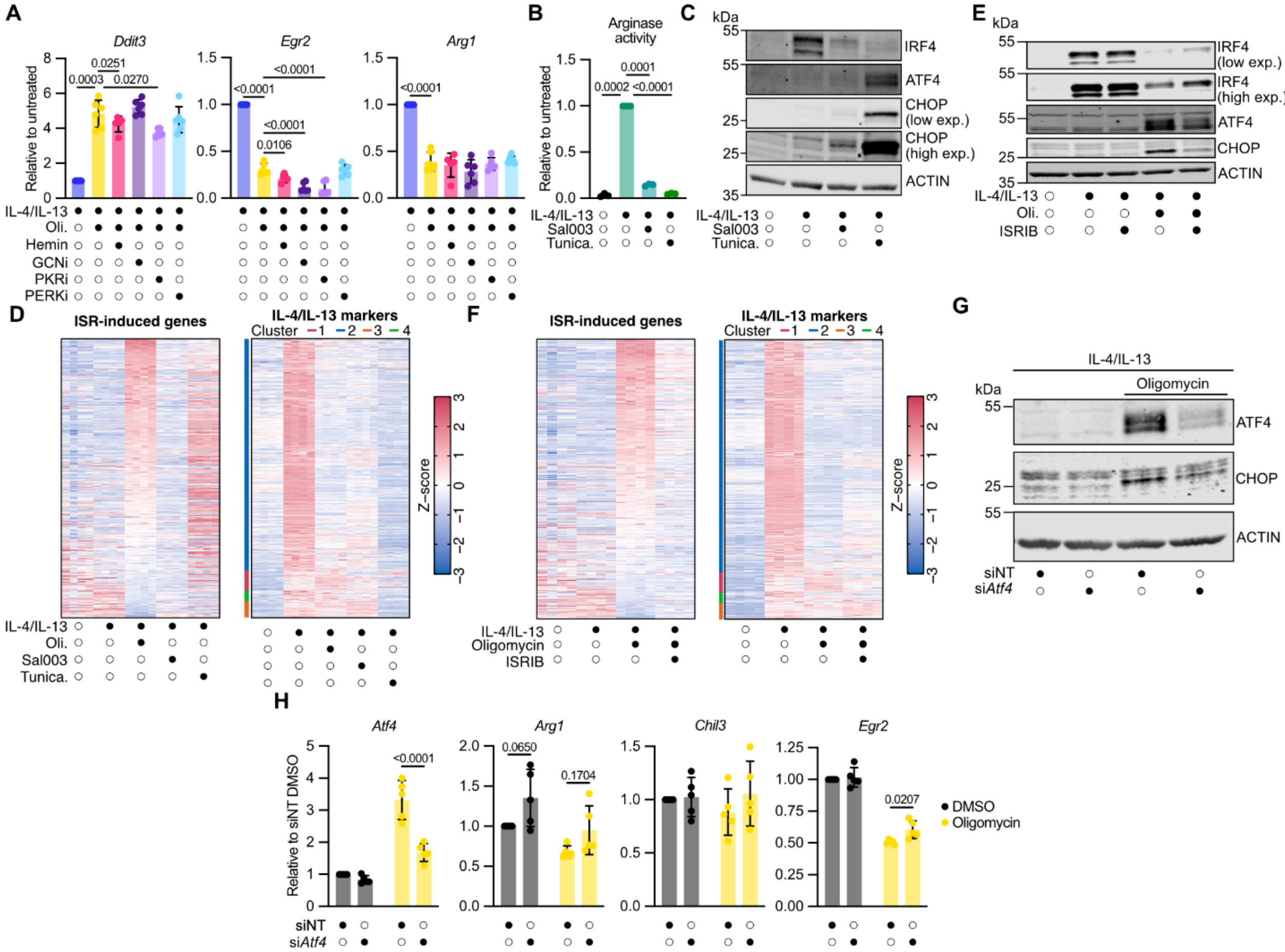

Supplemental Figure 5. ISR activation blocks IL-4/IL-13 macrophage activation. **(A)** qPCR analysis of ISR- (*Ddit3*) and IL-4/IL-13-induced (*Egr2* and *Arg1*) transcripts in 24 h IL-4/IL-13 macrophages treated with oligomycin (Oli.) in the presence of specific inhibitors of the four different ISR kinases. **(B)** Relative arginase activity (N = 6 mice from 2 independent experiments) and **(C)** representative immunoblot analysis of IRF4, ATF4, and CHOP levels in 24 h IL-4/IL-13-stimulated macrophages treated with ISR activators tunicamycin (Tunica.) and Sal003. **(D)** Expression levels of (left) ISR-induced genes and (right) IL-4/IL-13 markers from transcriptomics analysis of naive or 24 h IL-4/IL-13-stimulated BMDM treated with oligomycin and ISR activators (Sal003 and tunicamycin) (N = 4 mice). **(E)** Representative immunoblot analysis of IRF4, ATF4, and CHOP levels in 24 h IL-4/IL-13 BMDM treated with oligomycin and ISRIB, an ISR inhibitor. **(F)** Expression levels of (left) ISR-induced genes and (right) IL-4/IL-13 markers from transcriptomics analysis of naive or 24 h IL-4/IL-13-stimulated BMDM treated with oligomycin or oligomycin and the ISR inhibitor ISRIB (N = 4 biological replicates). **(G)** Representative immunoblot of ATF4 and CHOP in 24 h oligomycin-treated IL-4/IL-13 macrophages transfected with siRNA against *Atf4*. siNT: Non-targeting siRNA. **(H)** qPCR analysis of ISR kinase *Atf4* and IL-4/IL-13-induced (*Arg1*, *Chil3*, and *Egr2*) transcripts in 24 h oligomycin-treated IL-4/IL-13 macrophages transfected with siRNA against *Atf4* (N = 5 mice from three independent experiments). All data are shown as mean ± SD. Clusters from (D) and (F) are based on clusters defined on Fig. 3G. Paired one-way ANOVA with multiple comparisons to oligomycin-treated IL-4/IL-13 corrected with the Dunnett test for (A). Paired one-way ANOVA with multiple

comparisons to untreated IL-4/IL-13 corrected with the Dunnett test for (B). Paired two-way ANOVA with multiple comparisons to the respective siNT corrected with Šídák test for (H).

Data S1. (separate files)

Supplementary Table 1. Transcript per million (TPM) values of OXPHOS inhibitors RNA-seq datasets.
Supplementary Table 2. Proteomics analysis of whole cell and immunocaptured mitochondria.
Supplementary Table 3. RNA-seq derived list of activation markers.
Supplementary Table 4. GSEA of mouse gene ontology pathways in RNA-seq and proteomics datasets.
Supplementary Table 5. GSEA of a curated list of MitoCarta3.0 pathways in RNA-seq and proteomics datasets.
Supplementary Table 6. Activation markers clustering from RNA-seq with OXPHOS inhibitors.
Supplementary Table 7. DEseq2 analysis of ISR modulators RNA-seq datatsets.
